# Quality of life and mental health in breast cancer survivors compared with non-cancer controls: a study of patient-reported outcomes in the United Kingdom

**DOI:** 10.1007/s11764-020-00950-3

**Published:** 2020-10-21

**Authors:** Helena Carreira, Rachael Williams, Harley Dempsey, Susannah Stanway, Liam Smeeth, Krishnan Bhaskaran

**Affiliations:** 1 Department of Non-Communicable Disease Epidemiology, Faculty of Epidemiology and Population Health, London School of Hygiene & Tropical Medicine, Keppel street, London, WC1E 7HT UK; 2 Clinical Practice Research Datalink (CPRD), Medicines and Healthcare products Regulatory Agency, 10th Floor, 10 South Colonnade, Canary Wharf, London, E14 4PU UK; 3 Department of Medicine, The Royal Marsden NHS Foundation Trust, Fulham Road, London, SW3 6JJ UK; 4 Department of Medicine, The Royal Marsden NHS Foundation Trust, Downs Road, Surrey SM2 5PT Sutton, UK

**Keywords:** Breast cancer, Anxiety, Depression, Quality of life, United Kingdom

## Abstract

**Purpose:**

There is limited high-quality evidence on quality of life, anxiety, and depressive symptoms in breast cancer survivors and women with no history of cancer. We aimed to address this by comparing patient-reported outcomes between breast cancer survivors and women with no history of breast cancer*.*

**Methods:**

Breast cancer survivors and women with no prior cancer were selected from the UK Clinical Practice Research Datalink GOLD primary care database, which includes population-based primary care electronic health record data. Breast cancer survivors and controls were frequency matched by age and primary care practice. Outcomes were assessed with validated instruments via postal questionnaire. Linear and logistic regression models were fitted to estimate adjusted associations between breast cancer survivorship and outcomes.

**Results:**

A total of 356 breast cancer survivors (8.1 years post diagnosis) and 252 women with no prior cancer participated in the study. Compared with non-cancer controls, breast cancer survivors had poorer QoL in the domains of cognitive problems (adjusted *β (aβ) = 1.4, p = 0.01*), sexual function (a*β = 1.7, p = 0.02*) and fatigue (a*β = 1.3, p = 0.01*), but no difference in negative feelings, positive feelings, pain, or social avoidance. Breast cancer survivors had higher odds of borderline-probable anxiety (score *≥ 8*) (adjusted OR = 1.47, 95%CI:1.15–1.87), but no differences in depression. Advanced stage at diagnosis and chemotherapy treatment were associated with poorer QoL.

**Conclusions:**

Compared with women with no history of cancer, breast cancer survivors report more problems with cognition, sexual function, fatigue, and anxiety, particularly where their cancer was advanced and/or treated with chemotherapy.

**Implications for Cancer Survivors:**

Breast cancer survivors with more advanced disease and/or treated with chemotherapy should be closely monitored and, when possible, offered evidence-based intervention for fatigue, cognitive dysfunction, and sexual problems.

**Electronic supplementary material:**

The online version of this article (10.1007/s11764-020-00950-3) contains supplementary material, which is available to authorized users.

## Introduction

Millions of women worldwide are living beyond breast cancer, including over 2.7 million in the USA and 500,000 in the United Kingdom (UK) [1, 2]. Numbers are projected to rise further due to increasing trends in both incidence and survival [2], which demands for a better understanding of the long-term consequences of having a history of breast cancer.

Many women undergoing treatment for recently diagnosed breast cancer report clinically relevant symptoms of anxiety and/or depression [3, 4], and impairments for virtually all domains of quality of life (QoL) [5, 6]. Longitudinal studies have shown that mental health symptoms and QoL scores tend to improve over time, with many breast cancer survivors reaching similar levels to those of the general population around 1 year after the diagnosis [7, 8]. However, specific groups of breast cancer survivors appear to continue to have poorer QoL, anxiety, and depression in the long-term [9]. Factors that have been associated with poorer mental health and quality of life include younger age at diagnosis [10, 11], lower socio-economic status [12], persistent fatigue [13], lymphedema or arm symptoms [14, 15], and having had chemotherapy [16, 17].

The UK National Cancer Research Institute identified several areas related to psychological wellbeing and QoL in the top 20 priorities for patients living with and beyond cancer [18], but research on the QoL of breast cancer survivors in the UK is scarce [19–21]. A large study on the QoL of breast, colorectal, and prostate cancer survivors (5 to 16 years post diagnosis) reported that a proportion of cancer survivors in the UK may have poorer long-term QoL and need of support [21], and highlighted the need to identify these patients’ characteristics.

This study aimed to quantify patient-reported outcomes of QoL, anxiety, and depressive symptoms, in breast cancer survivors (> 1 year) compared with women with no prior history of cancer, and investigate socio-demographic and clinical determinants of QoL and mental health among breast cancer survivors.

## Methods

### Study design, sample size, and sampling frame

We designed a matched cross-sectional study including breast cancer survivors and a comparison group of women with no prior cancer. Between October 2018 and August 2019, we invited all primary care practices contributing with data to the Clinical Practice Research Datalink (CPRD) General Practitioners Online Database (GOLD) to participate in the study. CPRD is a UK government research service that collects, processes, and releases anonymised electronic health records from patients attending the UK National Health Service [22]. Patients registered with primary care practices that accepted to participate were potentially eligible for the study (see details below).

### Patient eligibility criteria

Inclusion criteria for the group of breast cancer survivors were as follows: (1) a diagnosis of invasive breast cancer (all stages) at least 1 year before, (2) aged 18–80 years, and (3) at least 1 year of follow-up in CPRD prior to the diagnosis (to ensure that the cancer was incident). For the comparison group, inclusion criteria were (1) no history of cancer (except non-melanoma skin cancer), (2) age 18–80 years, and (3) at least 2 years of follow-up data in CPRD (since we required 1 year of follow-up before and after cancer in the exposed group). Exclusion criteria for both groups were (1) inability to complete a self-reported questionnaire (e.g. due to dementia) and (2) having had another (non-breast) cancer or having been treated for a non-invasive breast tumour.

### Patient selection and recruitment

The CPRD GOLD primary care database was used to identify all breast cancer survivors from the participating practices. Women in the comparison group were randomly selected from the same practice, and frequency-matched on age to the breast cancer survivors in that practice. Initially controls were matched to breast cancer survivors with a ratio of 1:1, but this was revised early during recruitment to 2:1 due to ~ 50% lower response among controls. General practitioners reviewed the records of potentially eligible patients, applied inclusion/exclusion criteria (*vide* above), and sent the study materials to the eligible patients’ addresses with a pre-paid envelope to return the questionnaires. Patients were recruited between January and October 2019.

### Patient-reported outcomes

QoL was assessed with the Quality of Life in Adult Cancer Survivors (QLACS) scale [23]. This tool includes 47 items referring to the previous 4 weeks, divided in 7 generic domains (i.e. negative feelings, positive feelings, cognitive problems, pain, sexual function/interest, energy/fatigue, and avoidance) and 5 cancer-specific domains (i.e. financial problems, benefits of cancer, distress-family, appearance, distress-recurrence). Women in the comparison group replied to the generic domains only.

Anxiety and depressive symptoms were measured with the Hospital Anxiety and Depression Scale (HADS) [24]. This is a 14-item self-reported screening tool for anxiety and depressive symptoms in the past week [24]. The recommended cut-offs were used to categorise patients as non-case (scores 0–7), borderline (scores 8–10), and probable case (scores 11–21) [24].

### Socio-demographic and clinical information

All women were asked to complete a questionnaire with information on education, ethnicity, and social support through proxy of living arrangements (alone/not alone). Breast cancer survivors provided information about cancer treatments, stage of the disease at diagnosis, menopausal status, and status of the disease (active/remission). Patient-reported information was linked to the patient’s electronic health record and practice-postcode quintile of Index of Multiple Deprivation (IMD).

### Statistical analysis

#### Descriptive QoL and anxiety and depressive symptoms

We calculated QoL-domain scores for each patient. When there was one missing response for an item within a domain, we imputed the mean of the responses to the other items within that domain; if ≥ 2 responses were missing, the domain score was not calculated [25]. The summary score for generic domains of QoL was calculated by the sum of the domain scores, with reverse scoring for positive feelings. For the summary cancer specific domain, we added all domain scores except ‘benefits from cancer’. We calculated mean scores for each sub-scale of HADS. When there were three or fewer items missing per sub-scale, we imputed the average of the responses in that subscale [26]. Scores were summarised for each group using means and measures of dispersion.

#### Comparison of outcomes between breast cancer survivors and controls

We fitted domain-specific multiple linear regression models, using the domain scores as the dependent variable and the following independent variables: patient group (exposed vs. control), age group (< 60, 60–69, ≥ 70 years), higher education degree (yes/no), and quintile of IMD. Interactions between the exposure and socio-demographic variables were tested but not included in the final models as these were not significant.

Outcome-specific logistic regression models were used to estimate the association between breast cancer survivorship and probable anxiety (HADS-A ≥ 11) and depression (HADS-D ≥ 11). Models were further adjusted for age (< 60, 60–69, ≥ 70 years), higher education degree (yes/no), and quintile of IMD. In an a priori defined sensitivity analysis (see study protocol, Supplementary file), we used a lower cut-off (HADS-A ≥ 8; HADS-D ≥ 8) for caseness, as the standard cut-off ≥ 11 was found to have low sensitivity (50%, 95%CI 27% to 73%) to detect cases of depression in this patient population [27].

For all models, robust standard errors were computed to account for patient clustering by primary care practice, and regression coefficients (*β*) and 95% confidence intervals (95%CI) were reported.

#### Socio-demographic and clinical determinants of QoL, and anxiety and depressive symptoms in breast cancer survivors

We used linear regression models to assess the impact of socio-demographic, clinical, and treatment factors on the generic and cancer-specific domains of QoL, and HADS-subscales. Socio-demographic variables were age (< 60, 60–69, and ≥ 70 years), practice postcode-linked IMD quintile, higher education degree (yes/no), and living arrangements (alone/not alone). Clinical variables were type of surgery (breast conserving/mastectomy), breast reconstruction (yes/no), stage at diagnosis (localised/lymph node or distant metastases), remission status (yes/no), menopausal status (pre/postmenopausal), time since diagnosis (1–5, 5–10, and > 10 years), and treatment with chemotherapy (yes/no), radiotherapy (yes/no), hormone therapy (yes/no), and immunotherapy (yes/no). For age at diagnosis, education, stage at diagnosis, and exposure to chemotherapy, we fitted models adjusted for socio-demographic factors only (age, education, IMD quintile and country), as well as models adjusted for chemotherapy (yes/no) and stage at diagnosis (early/advanced). The regression coefficients (*β*) and respective 95%CIs were reported.

## Results

**Fig. 1:**
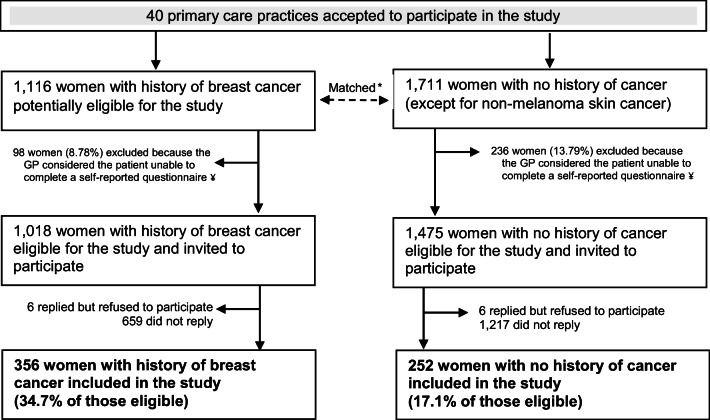
Flowchart of patient recruitment. CPRD Clinical Practice Research Datalink. *Women in the comparison group included a random sample of women with no history of cancer that had the same age distribution as the breast cancer survivors in the primary care practice (frequency matching). Initially matching occurred on a ratio of one case to one control. We later revised this to one case to two controls, to account for lower participation rate in the control group. ¥Exclusion criteria included patients with dementia, terminally ill, or with another cancer diagnosis. GPs also excluded patients who were not able to complete questionnaires in English, or who had died or transferred out of their practice recently

A total of 356 breast cancer survivors and 252 women with no history of cancer, from 40 primary care practices in the UK, participated in the study (Fig. 1). Participants tended to live in more affluent areas compared with non-participants (Supplementary Table 1). Mean age was 64.8 years among breast cancer survivors (standard deviation (SD) = 9.0; range 34–81) and 65.5 years in the non-cancer comparison group (SD = 9.4; range 36–81). In both groups, a quarter of the women had a higher education degree (Table 1). Breast cancer survivors were on average 8.1 years post diagnosis (SD = 4.6; range 2–26). About 99% of the breast cancer survivors had surgery (35% mastectomy), 80% radiotherapy, 49% hormone therapy, and 41% chemotherapy. Most women had been diagnosed with localised (54.4%) or locally invasive disease (43.3%). Table 2 shows the mean scores for QoL domains in the two groups.

**Table 1:** Characteristics of the study participants*§

	No history of cancer (*N* = 252)		Breast cancer survivors (*N* = 356)	
	*N*	%	*N*	%
Age at completion of questionnaire				
34–59 years	71	28.1	103	28.9
60–69 years	80	31.7	130	36.5
≥ 70 years	101	40.1	123	34.6
Highest education level (schooling years)				
Up to GCSEs, O levels, or equivalent (Up to 11 years)	78	31.0	127	35.7
A levels or equivalent (13 years)	29	11.5	36	10.1
Trade or technical training	52	20.6	54	15.2
University degree (>13 years)	66	26.2	94	26.4
Did not want to disclose	27	10.7	45	12.6
Ethnicity				
White	242	96.0	347	97.5
Asian / Asian British	6	2.4	1	0.3
Did not want to disclose	4	1.6	8	2.3
IMD quintile				
1 (least deprived)	53	21.0	71	19.9
2	36	14.3	54	15.2
3	26	10.3	55	15.4
4	98	38.9	141	39.6
5 (most deprived)	39	15.5	35	9.8
Living arrangements				
Not alone	185	73.4	273	76.7
Alone	63	25.0	75	21.1
Did not want to disclose	4	1.6	8	2.2
Country				
England	64	25.4	50	14.0
Northern Ireland	16	6.3	33	9.3
Scotland	74	29.4	114	32.0
Wales	98	38.9	159	44.7
Time since breast cancer diagnosis				
1–5 years	-	-	134	37.6
5–10 years	-	-	113	31.7
10–15 years	-	-	88	24.7
15–20 years	-	-	16	4.5
> 20 years	-	-	5	1.4
Breast cancer treatments				
Surgery	-	-	351	98.6
Lumpectomy	-	-	226	63.5
Mastectomy	-	-	125	35.1
Reconstruction	-	-	44	12.4
Radiotherapy	-	-	283	79.5
Chemotherapy	-	-	145	40.7
Hormone therapy	-	-	175	49.2
Immunotherapy	-	-	6	1.7
Stage at diagnosis				
Localised to the breast	-	-	195	54.8
Regional metastases	-	-	153	43.0
Distant metastases	-	-	2	0.6
Unknown	-	-	6	1.7
Time since last treatment for breast cancer				
Under treatment	-	-	3	0.8
< 12 months	-	-	2	0.6
Between 1 and 5 years	-	-	124	34.8
More than 5 years	-	-	219	61.5
Does not know	-	-	8	2.2
Disease status at questionnaire response				
In remission	-	-	322	90.4
Active disease	-	-	7	2.0
Does not know			27	7.6
Menopausal status				
Menopausal at breast cancer diagnosis	-	-	244	68.5
Became menopausal during breast cancer treatment	-	-	72	20.2
Not menopausal	-	-	33	9.3
Unknown	-	-	7	2.0

*Information on age at questionnaire completion, time since breast cancer diagnosis, practice postcode IMD quintile, and country were obtained from the electronic health records of the participating patients. Information on education, ethnicity, living arrangements, treatments for breast cancer, stage at diagnosis, time since last treatment for breast cancer, and disease and menopausal status were collected directly from the patients using a self-reported questionnaire

§Proportion may not add to 100% due to rounding

**Table 2:** Quality of life anxiety and depressive symptoms, in breast cancer survivors and in women who never had cancer

	Women with no history of cancer (N = 252)						Women with history of breast cancer (N = 356)					
	No. (No. imputed*)	Mean score	SD	Range	% Floor^¶^	% Ceiling^¶^	No. (No. imputed*)	Mean score	SD	Range	% Floor	% Ceiling
Quality of life in adult cancer survivors§												
Generic												
Negative feelings	251 (7)	11.0	4.9	4–26	3.7	0.0	346 (22)	11.6	5.3	4–28	4.3	0.3
Positive feelings^§^	250 (5)	21.1	5.4	7–28	0.0	9.9	347 (18)	20.8	5.7	7–28	0.0	10.7
Cognitive problems	251 (4)	10.5	4.7	4–28	6.2	0.8	350 (19)	11.7	5.3	4–27	4.9	0.0
Physical pain	249 (4)	11.1	6.5	4–28	12.0	0.8	347 (19)	11.2	6.2	4–28	9.5	1.4
Sexual interest/function	227 (8)	12.0	6.4	4–28	13.2	2.7	330 (27)	13.6	7.2	4–28	13.0	4.9
Fatigue/Energy	251 (1)	12.3	4.8	4–24	4.1	0.0	350 (11)	13.3	5.0	4–25	3.7	0.0
Social avoidance	251 (21)	9.8	5.6	4–28	17.3	0.4	347 (45)	9.9	5.8	4–28	24.8	0.6
Summary ‡	226 (0)	75.7	27.7	28–157	–	–	318 (0)	80.4	29.0	29–162	-	-
Cancer-specific												
Financial problems	-	-	-	-	-	-	351 (9)	7.3	5.2	4–28	46.2	0.6
Distress related to family	-	-	-	-	-	-	352 (4)	12.3	7.5	4–28	2.6	7.8
Appearance concerns	-	-	-	-	-	-	350 (12)	9.4	5.9	4–28	20.2	4.8
Distress over recurrence	-	-	-	-	-	-	351 (8)	13.9	6.9	4–28	27.4	0.9
Summary	-	-	-	-	-	-	350 (0)	42.7	19.6	16–106	7.1	2.9
Benefits of cancer^§^	-	-	-	-	-	-	348 (7)	17.0	6.6	4–28	-	-
Hospital Anxiety and Depression Scale												
Anxiety	248 (7)	6.4	4.1	0–20	5.0	0.4	351 (4)	6.8	4.6	0–20	8.0	0.3
Depression	249 (4)	3.6	3.3	0–17	14.5	0.0	352 (3)	3.6	3.6	0–19	17.6	0.0

*SD* standard deviation

*Number of patient with score imputed. When one item was missing out of the four items in the domain, we imputed this item with the arithmetic mean of the values in the other three items. Mean domain score was not calculated for patients that did not reply to two or more items in a domain

§Higher scores represent poorer QoL, except for the domains ‘positive feelings’ and ‘benefits of cancer’

‡Calculated as the sum of all domain scores except for positive feelings

^¶^Proportion of patients with domain scores at the extreme low (floor) or high (ceiling) of the distribution

### QoL, anxiety, and depressive symptoms in breast cancer survivors compared with women with no history of cancer

After adjusting for age, education, and deprivation, breast cancer survivors had poorer QoL compared with controls for cognitive problems (adjusted *β* (*aβ*) = 1.4; 95%CI 0.4–2.3, *p*=0.01), sexual function (*aβ* = 1.7; 95%CI 0.4–3.1, *p*=0.02), and fatigue (*aβ* = 1.3; 95%CI 0.4–2.2, *p*=0.01) (Table 3); differences for other domains were compatible with chance variation. Compared with controls, breast cancer survivors treated with chemotherapy reported significantly poorer overall quality of life, with more negative feelings, cognitive problems, sexual dysfunction, fatigue, and anxiety (Table 3). Similar results were found for breast cancer survivors diagnosed with more advanced disease, except for anxiety scores that were not significantly different between groups.

**Table 3:** Comparison of patient reported outcomes between breast cancer survivors and controls, by chemotherapy and stage at diagnosis

	Women with no history of cancer	Women with a history of breast cancer									
		All		Treated with chemotherapy				Stage of disease at diagnosis			
				No chemotherapy		Chemotherapy		Localised		Advanced	
		β*	95%CI	β*	95%CI	β*	95%CI	β*	95%CI	β*	95%CI
QLACS:											
Generic domains											
Negative feelings	Ref.	0.7	− 0.1–1.4	0.1	− 0.7–1.0	1.4	0.2–2.6	0.2	− 0.6–1.0	1.4	0.2–2.5
Positive feelings	Ref.	− 0.3	− 1.2–0.6	− 0.2	− 1.2–0.8	− 0.6	− 1.9–0.7	− 0.2	− 1.2–0.7	− 0.5	− 1.8–0.8
Cognitive problems	Ref.	1.4	0.4–2.3	0.6	− 0.3–1.5	2.6	1.3–3.8	0.9	− 0.1–1.9	2.0	0.9–3.2
Pain	Ref.	0.2	− 1.1–1.6	− 0.2	− 1.6–1.2	0.9	− 0.8–2.6	− 0.6	− 2.0–0.8	1.2	− 0.4–2.8
Sexual function	Ref.	1.7	0.4–3.0	1.2	0.0–2.4	2.5	0.6–4.3	0.8	− 0.5–2.1	2.9	1.3–4.6
Energy/Fatigue	Ref.	1.3	0.4–2.2	1.2	0.2–2.1	1.5	0.4–2.7	1.0	0.0–2.0	1.7	0.6–2.9
Avoidance	Ref.	0.1	− 1.0–1.2	− 0.3	− 1.5–0.9	0.8	− 0.6–2.2	− 0.2	− 1.2–0.8	0.6	− 0.9–2.2
Summary	Ref.	5.9	− 0.2–11.8	2.3	− 3.6–8.2	11.0	3.6–18.3	1.8	− 4.1–7.7	11.1	3.8–18.3
HADS											
Anxiety	Ref.	0.5	− 0.1–1.0	0.1	− 0.6–0.8	1.1	0.2–2.0	0.4	− 0.3–1.0	0.8	− 0.2–1.7
Depression	Ref.	0.1	− 0.5–0.7	0.1	− 0.6–0.7	0.2	− 0.7–1.0	0.0	− 0.7–0.7	0.3	− 0.5–1.0

*Beta scores represent the difference between the scores of breast cancer survivors and the scores of the women in the comparison group. These were adjusted for adjusted for age, education, and IMD quintile. Positive beta scores indicate more problems (poorer QoL) in breast cancer survivors

*HADS* Hospital Anxiety and Depression Scale, *QLACS* Quality of Life in Adult Cancer Survivors, *95%CI* 95% confidence interval

Breast cancer survivors had non-significantly higher odds of probable anxiety (HADS-anxiety score ≥ 11) than controls (adjusted odds ratio (aOR) = 1.37, 0.95–1.97; Table 4). However there was strong evidence of a difference when a more sensitive threshold (score ≥ 8, ‘borderline/probable anxiety’) was used (aOR = 1.46, 1.14–1.88). There were no differences in odds of probable depression (aOR = 1.17, 0.52–2.67).

**Table 4:** Unadjusted and adjusted associations between breast cancer survivorship and anxiety and depression categories

	Anxiety						Depression					
	Cut-off ≥ 11 for caseness (probable anxiety)			Cut-off ≥ 8 for caseness (borderline/probable anxiety)			Cut-off ≥ 11 for caseness (probable depression)			Cut-off ≥ 8 for caseness (borderline/probable depression)		
	No. of cases (%)	OR	95%CI	No. of cases (%)	OR	95%CI	No. of cases (%)	OR	95%CI	No. of cases (%)	OR	95%CI
Unadjusted												
No cancer	44 (17.4)	Ref		87 (35.1)	Ref		9 (3.6)	Ref		38 (15.3)	Ref	
Breast cancer	80 (22.8)	1.37	0.95–1.97	155 (44.2)	1.46	1.14–1.88	17 (4.8)	1.35	0.68–2.68	54 (15.3)	1.00	0.72–1.41
Adjusted*												
No cancer	39 (17.7)	Ref		79 (35.8)	Ref		8 (3.6)	Ref		33 (14.9)	Ref	
Breast cancer	70 (22.7)	1.41	0.93–2.13	139 (45.1)	1.48	1.16–1.89	12 (3.9)	1.17	0.52–2.67	46 (14.9)	1.07	0.77–1.50

*Adjusted for age, education, and IMD quintile in multivariate analyses. The number of patients in multivariate analyses varies due to missing data for education level

*OR* odds ratio, *95%CI* 95% confidence interval

### Determinants of QoL and symptoms of anxiety and depression in breast cancer survivors

**Fig. 2:**
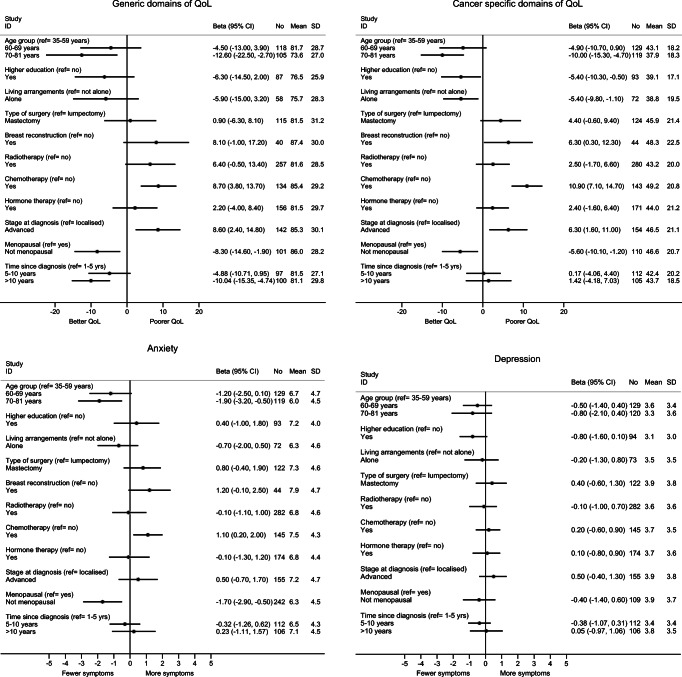
Quality of life, and anxiety and depressive symptoms, in breast cancer survivors, by socio-demographic, clinical, and treatment characteristics (*N* = 356)

Figure 2 shows the variation of the summary scores of QoL, anxiety, and depression, by socio-demographic and clinical variables. For both generic and cancer-specific domains of QoL, younger age at questionnaire response, chemotherapy treatment, lymph node involvement, and not being menopausal at diagnosis were all associated with poorer QoL. The associations remained statistically significant after adjusting for socio-demographic variables, except for the effect of menopause on the generic domains of QoL, which was no longer statistically significant (Supplementary Tables 2, 3, 4 and 5). The effects of chemotherapy, menopausal status, and stage at diagnosis were no longer statistically significant after further adjusting for treatment variables. Higher education degree and not living alone were associated with better QoL for the cancer specific domains, but only education remained significantly associated after adjusting for socio-demographic and clinical variables (Supplementary Tables 6 and 7). Increasing time since diagnosis resulted in better QoL for the generic domains, while for the cancer-specific domains no variation on QoL was noted by proximity to diagnosis.

For individual QoL domains, in fully adjusted models, older women (≥ 70 years) had significantly fewer negative feelings, cognitive problems, sexual dysfunction, avoidance, financial problems, and family- and recurrence-related distress compared with younger breast cancer survivors (< 60 years) (Supplementary Table 2). Pre-menopausal women at diagnosis reported more negative feelings and avoidance compared with those who were menopausal (Supplementary Table 3). Women treated with chemotherapy had more cognitive problems, and appearance and recurrence concerns compared with those that did not receive chemotherapy (Supplementary Table 4). Compared with women diagnosed with localised disease, those with lymph node or distant metastases reported more pain and sexual dysfunction (Supplementary Table 5). Women living alone reported more concerns with cancer recurrence, while women with higher education had significantly less pain and distress related to their family members getting cancer (Supplementary Tables 6 and 7).

Anxiety and depression were lower in women ≥ 70 years old, compared with those < 60 years, and this remained significant after control for confounding. Anxiety was also significantly increased in women treated with chemotherapy and women not menopausal at diagnosis, but only the latter remained statistically significant upon further adjustments.

## Discussion

Breast cancer survivors had more cognitive problems, sexual dysfunction, fatigue, and borderline to abnormal anxiety symptoms than women who did not have cancer. The increased risk was driven by exposure to chemotherapy and more advanced disease at diagnosis. Among breast cancer survivors, younger age, no higher education degree, pre-menopausal status and more advanced disease at diagnosis, and treatment with chemotherapy were all associated with poorer QoL. Younger age was also associated with increased anxiety and depressive symptoms.

This study has several strengths. We uniquely combined the strengths of routinely collected electronic health records data, enabling us to identify a large population-based sample of breast cancer survivors and controls, with direct and targeted data collection using validated instruments. We selected patients from the CPRD GOLD primary care database, which is representative of the UK population in terms of age, sex, and ethnicity [28]. Matching groups by primary care practice and age is likely to have accounted for measurable and some immeasurable confounding; we further collected data for education, ethnicity, and a proxy of social support, which are known to be imperfectly recorded in the patients’ clinical records, and this allowed us to account for these variables in analyses. The validity of the tools used to assess outcomes has been established [29–32]. Finally, our study was sufficiently powered for the main comparison of QoL between breast cancer survivors and controls, as we exceed the target sample size. However, this study also has limitations. The major threat to the validity of our results comes from the low participation rate (35% in the breast cancer survivors group; 17% in the control group). Even though this participation rate overall surpassed our estimate at study design of 20%, and is similar to participation rates in QoL studies in cancer survivors in the UK [33], we cannot rule out selection bias where healthier women, both physically and psychologically, are more likely to participate. Our final sample overwhelmingly included white women, without distant metastases, and not undergoing further treatment for breast cancer (excluding tamoxifen), and thus, our results may not be generalisable to all breast cancer survivors. Another limitation is that clinical information was self-reported, which may have led to some information bias, but we expect this to have minor impact on our results. The QLACS was well accepted but some missing responses were observed, most often for items related to sexual interest and function, and social avoidance; our proportion of missing data was similar to another study assessing QoL with QLACS in cancer survivors in the UK [29]. It is unclear whether the missing responses were related to values themselves, but it is plausible that women with sexual problems may not feel comfortable in reporting their sexual function, despite the anonymous nature of the data collection. In addition, older women may have fewer opportunities to engage in partnered sexual activity (e.g. widowed, erectile dysfunction in partners), and therefore consider these items not applicable to them. For the social avoidance domain, one item was particularly more often not replied—related to being ‘reluctant to start new relationships’. We think this item might have been interpreted by the patients as starting new romantic relationships, and thus left blank due to no applicability. Lastly, residual confounding may be present in our results, particularly for socio-economic status, which we measured by proxy of education and quintile of Index of Multiple Deprivation.

Similarly to studies conducted in the UK and elsewhere [11, 21, 34–36], our results show that only a subgroup of breast cancer survivors have poor QoL. While previous studies in the UK focused in survivors > 5 years after diagnosis, we uniquely included patients from the 1st anniversary of their cancer diagnosis and identified the characteristics associated with poorer QoL; these can be used to inform allocation of resources, but caution must be taken to not overgeneralise our results. For example, Dialla et al. [37] reported that disease progression was among the most important determinants of poor QoL among younger women; we had very few patients with active disease, and therefore were not able to assess this. Our results were also consistent with previous studies addressing the links between breast cancer survivorship and sexual dysfunction, fatigue, and exposure to chemotherapy and worse cognitive function [38–42]. The poorer QoL and mental health in younger breast cancer survivors is likely to be explained by specific concerns that may not be applicable to older women, such as fertility and body image issues and/or the impact of their possible death for their young offspring [10, 16, 17, 43]. Post-traumatic growth, the phenomenon in which women appreciate life more after a traumatic event [44], is likely explain the better QoL of older women as they also had the highest scores for positive feelings and benefits of cancer.

Public health interventions to improve QoL ought to be comprehensive, including risk reducing and reactive strategies. Studies have reported that eight in 10 women with breast cancer in the UK were not told about the potential long-term impact of the cancer on their mental health [45], and 41% of women reported not having received the professional support needed to cope with the long-term consequences of their disease [46]. Patient education for prevention and early detection of treatment-related sequelae should start as early as possible (in the pre-operative period), focusing on both physical and mental health [47, 48]. When survivors report issues related to QoL, interventions should be based in evidence. A review of systematic reviews of non-pharmacological interventions to improve QoL in breast cancer survivors found that exercise interventions, yoga, and other physical activity-related interventions, cognitive-behavioural therapy, and mindfulness-based stress reduction all were beneficial for the patients’ quality of life [49]. However, access to these interventions depends largely on availably, which is known to be currently insufficient [46, 50]. Where these are available, raising awareness about the services locally available to patients, and how to access them, cannot be overlooked (e.g. Breast Cancer Now, a UK-based charity, has created a course tailored to breast cancer survivors, which was found to improve patients’ HRQoL, emotional wellbeing, and self-management measures [51]). On the pharmacological side, it is also important to raise awareness among health care professionals of the importance of addressing issues such as sexual function, which is a topic often poorly communicated in clinics [52, 53].

Future research on the QoL of breast cancer survivors in the UK should focus on the barriers patients face when accessing interventions aimed at preventing declines in QoL in the long term, as well as assessing time trends in QoL, as it is unclear if modern treatments yield better QoL. Evidence on non-pharmacological interventions for QoL in cancer survivors tended to include patients in the first few years of their survivorship journey [49]; future studies need to establish the efficacy of interventions in long-term survivors. Studies are also needed to assess if women diagnosed with breast carcinomas in situ have differ in terms of QoL from both breast cancer survivors and women who did not have cancer, as these tumours are treated similarly to early stage breast cancer.

In conclusion, breast cancer survivors in the UK reported higher risk of problems with cognition, sexual function, fatigue, and borderline/probable anxiety, particularly where their cancer was advanced and/or treated with chemotherapy. This information can be used to tailor increased surveillance for mental health and QoL issues in these groups.

## Electronic supplementary material

ESM 1 (DOCX 622 kb)
