# Supplementary material for: Quality of life and mental health in breast cancer survivors compared with non-cancer controls: a study of patient-reported outcomes in the United Kingdom
Source: J Cancer Surviv. 2020 Oct 21;15(4):564–75. doi: 10.1007/s11764-020-00950-3 (PMC8272697; doi:10.1007/s11764-020-00950-3)
Supplement: Supplementary file 1 — (DOCX 622 kb) [file 11764_2020_950_MOESM1_ESM.docx]

**Supplementary file**

Contents

[Study protocol mental health and quality of life of female breast cancer survivors compared to women who did not have cancer 2](#_Toc43565690)

[Supplementary table 1 Comparison between participants and non-participants by exposure group, age group, country, and deprivation. 42](#_Toc43565727)

[Supplementary table 2 Associations between age and quality of life, anxiety and depression in breast cancer survivors (N=353). 43](#_Toc43565728)

[Supplementary table 3 Associations between menopausal status at cancer diagnosis and quality of life, anxiety and depression in breast cancer survivors (N=353). 44](#_Toc43565729)

[Supplementary table 4 Associations between exposure to chemotherapy and quality of life, anxiety and depression in breast cancer survivors (N=353). 45](#_Toc43565730)

[Supplementary table 5 Associations between stage of the disease at diagnosis and quality of life, anxiety and depression in breast cancer survivors (N=353). 46](#_Toc43565731)

[Supplementary table 6 Associations between living arrangements and quality of life, anxiety and depression in breast cancer survivors (N=353). 47](#_Toc43565732)

[Supplementary table 7 Associations between education and quality of life, anxiety and depression in breast cancer survivors (N=353). 48](#_Toc43565733)


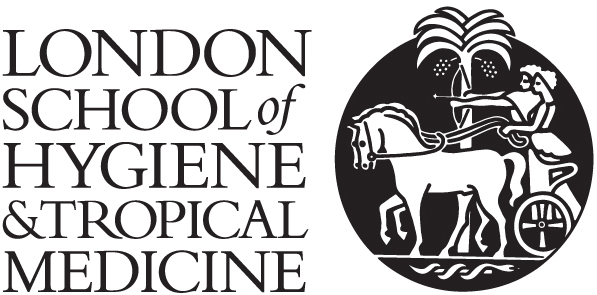


# **Study protocol: mental health and quality of life of female breast cancer survivors compared to women who did not have cancer**

**Research team**

Helena Carreira, London School of Hygiene & Tropical Medicine

Rachael Williams, Clinical Practice Research Datalink

Susannah Stanway, The Royal Marsden NHS Foundation Trust

Liam Smeeth, London School of Hygiene & Tropical Medicine

Krishnan Bhaskaran, London School of Hygiene & Tropical Medicine

London, May 2018

## Summary

We aim to assess the quality of life (QoL), and presence and severity of anxiety and depressive symptoms, in women who have had breast cancer diagnosed at ≥1 year, compared to women who did not have cancer.

The Clinical Practice Research Datalink (CPRD) primary care database will be used to select a random sample of breast cancer survivors (≥1 year), whose general practitioner (GP) agrees to participate in the study (see below), and who were registered with the practice for ≥1 year before and after the breast cancer diagnosis. Age-matched women who never had cancer will be randomly selected from the same practice. Staff at each practice will mail the study materials to the eligible women, who will complete the questionnaires and send those to the CPRD Intervention Studies Team for processing.

In addition, a secondary objective of this study is to assess whether PROs can be reasonably studied by using electronic health records (EHR), as these would involve fewer resources. For this, the EHR of the participating women will be collated from the CPRD primary care database and the results will be compared to those reported by the patients.

Table of Contents

Summary 3

1 Background 5

2 Aims and objectives 7

3 Plan of investigation 8

3.1 Study type 8

3.2 Study site 8

3.3 Study design 8

3.4 Study population 8

3.5 Comparison group 8

3.6 Recruitment of the participants 8

4 Data to be collected 11

4.1 Health-related quality of life 11

4.2 Anxiety and depression 11

4.3 Clinical and socio-demographic data 11

5 Data/statistical analysis 12

6 Plan for addressing missing data 19

7 Sample size 20

8 Feasibility counts 23

9 Pilot study 24

10 Limitations of the study design, data sources and analytical methods 25

11 Patient or user group involvement 26

12 Plans for disseminating and communicating study results 27

13 Timetable 28

14 References 29

15 Appendices 33

Appendix 1. List of read codes to identify breast cancer patients [50]. 34

Appendix 2. Items of the Quality of Life in Adult Cancer Survivors Scale (QLACS) grouped by domain 35

Appendix 3. Quality of Life in Adult Cancer Survivors Scale (QLACS) [36] 37

Appendix 4. Generic domains of HRQoL [36] 39

Appendix 5. Hospital Anxiety and Depression Scale [37] 41

Appendix 6. Clinical information 43

Appendix 7. Demographic information 45

## Background

Breast cancer is the most common malignancy diagnosed in women in the United Kingdom (UK), excluding non-melanoma skin cancer [[1](#_ENREF_1)]. The five-year age-standardised net survival for patients diagnosed with breast cancer in 2005-09 was 81% [[2](#_ENREF_2)]. Breast cancer survivors are the largest group of cancer survivors in the UK [[3](#_ENREF_3), [4](#_ENREF_4)]: approximately 570,000 women were estimated to be living with or beyond breast cancer in 2010; this corresponds to 1,803 per 100,000 women [[4](#_ENREF_4)]. The increasing trends in incidence and survival [[1](#_ENREF_1), [2](#_ENREF_2)] suggest that the number of breast cancer survivors will continue to increase in the next decades [[4](#_ENREF_4)].

Even though women now live longer after the breast cancer diagnosis, the disease is perceived as life threatening and a major cause of emotional distress [[5](#_ENREF_5)]. Common reactions to the diagnosis include anxiety, feelings of loneliness, fear of death, hopelessness, anger, suicidal thoughts and existential issues [[6](#_ENREF_6), [7](#_ENREF_7)]. In addition to the sorrow of the diagnosis, most women undergo a long and complex journey of aggressive treatments [[8](#_ENREF_8)] with iatrogenic effects that are likely to have a long-term negative impact on their mental health and health-related quality of life (HRQoL) [[9](#_ENREF_9), [10](#_ENREF_10)]. For example, surgery for tumour removal and lymph node status assessment may cause lymphoedema [[11](#_ENREF_11)] and/or persistent pain [[12](#_ENREF_12)], in addition to a life-long scar, which may change women’s body image [[13](#_ENREF_13)]. Chemotherapy may result in cognitive impairments [[14](#_ENREF_14), [15](#_ENREF_15)] and/or cause amenorrhea in pre-menopausal women, bringing fertility concerns (for women who want children) and vasomotor symptoms such as hot flushes, night sweats, breast sensitivity and/or pain [[16](#_ENREF_16), [17](#_ENREF_17)]. In the long-term, women also have to re-adapt to social and intimate relationships (including with their spouse [[18](#_ENREF_18)] and offspring [[19-21](#_ENREF_19)]), and deal with the fear of cancer recurrence and death [[22](#_ENREF_22)].

Patients often report the social, mental and cognitive functioning as important outcomes of their disease [[23-26](#_ENREF_23)]. However, few studies [[27](#_ENREF_27), 28] focused on the mental health and HRQoL of large samples of cancer survivors in the UK. The Clinical Practice Research Datalink (CPRD) primary care database gathers data for consultations occurring in a large number of general practices in the UK. This database currently includes data for more than 11.3 million patients, from over 600 general practices [[29](#_ENREF_29)]. The cohort of cancer survivors in this database is one of the largest in the world with data prospectively and routinely collected at primary care level. As most mental disorders are also managed at primary care level [[30](#_ENREF_30), [31](#_ENREF_31)], the CPRD primary care database offers a unique opportunity to study long-term mental disorders in women who have had breast cancer. The information available for some domains of HRQoL may also represent an opportunity to study what are normally patient reported outcomes at a much lower cost but there has been no study evaluating the extent to which EHR data can be reasonably used to study HRQoL.

Khan *et al* used the CPRD primary care data to evaluate the pattern of consultations for anxiety and depression in 2003-2005, as well as the prescription of antidepressants and anxiolytics, among 16,938 breast cancer survivors (>5 years) and 67,649 women without breast cancer [[27](#_ENREF_27)]. This study showed that breast cancer survivors had significantly increased odds of being prescribed antidepressants and anxiolytics but not of consulting for anxiety or depression, compared to women who did not have breast cancer [[27](#_ENREF_27)]. The interpretation of these results is not straightforward because: 1) patients consulting for anxiety or depression are likely to represent the most severe cases, as these disorders, especially in the sub-threshold or milder severities, are often undiagnosed [[31](#_ENREF_31)] and their burden underestimated; 2) cancer survivors may have more contact with the health services and be therefore more likely to be diagnosed and/or treated for anxiety or depressive symptoms, compared to women who did not have breast cancer; 3) antidepressants may also be prescribed to breast cancer survivors as treatment for hot flushes [[32](#_ENREF_32)], one of the commonest side effects of endocrine treatments [[33](#_ENREF_33)], and it is unclear if the frequency of prescription of antidepressants for hot flushes differs between women who have had breast cancer and women who never had cancer. Considering this, it is unclear how well the data registered in the EHR represent the burden of anxiety and depressive conditions in the population. In addition, a population-based cohort study conducted in Denmark described a significantly increased risk of depression in the first years after diagnosis, whose magnitude and significance reduced over time [[34](#_ENREF_34)]. Corresponding estimates for the five years after the diagnosis are not available in the UK.

The aim of this study is to investigate the HRQoL, and the presence and severity of anxiety and depressive symptoms, in breast cancer survivors (>1 year) and in women who did not have cancer. A secondary objective of this study is to compare the outcomes reported by the patients to the data available in the EHR. In doing so, we will assess the feasibility of using EHR to study outcomes that are usually reported directly by patients.

## Aims and objectives

Aims

The primary aim of this study is to investigate the health-related quality of life (HRQoL), and the presence and severity of anxiety and depressive symptoms, in female breast cancer survivors (>1 year) compared to women who did not have cancer.

The secondary aim is to assess the feasibility of studying outcomes that are usually reported directly be patients by relying on the EHR data.

Specific objectives

1. To describe cancer-specific measures of HRQoL in breast cancer survivors, and to explore the impact of demographic and clinical factors;
2. To compare measures of HRQoL between breast cancer survivors and women who did not have cancer and to evaluate the impact of clinical and demographic variables;
3. To compare the severity of anxiety and depressive symptoms in breast cancer survivors and in women who did not have cancer, and to assess the impact of demographic and clinical variables;
4. To compare patient reported HRQoL, and anxiety and depressive symptoms, with the information registered in the EHR for similar constructs.

## Plan of investigation

## Study type

Descriptive.

## Study site

England, Wales, Scotland and Northern Ireland.

## Study design

Cross-sectional.

## Study population

Women aged 18 to 80 years old, diagnosed with a first primary cancer of the breast at one year or more ago at the recruitment date, and who had been registered for at least two years with a general practice contributing with ‘up to standard’ data to CPRD at the moment of the recruitment.

## Comparison group

Adult women (18-80 years) without a previous cancer diagnosis, selected from the same primary care practices of the cancer patients.

## Recruitment of the participants

Participants will be recruited from primary care practices contributing with data to the CPRD primary care database, via their GP. GPs working in practices considered ‘active’ (i.e. contributing with data to CPRD at the time of recruitment), and whose data quality at practice level has been judged as ‘up to standard’ by the CPRD internal quality procedures, will be invited by the CPRD Intervention Studies Team to participate in the study. Refusal to participate in the study will be recorded.

Breast cancer survivors

The EHR of the women registered with the GPs who accept to participate in the study will be collated. We will create a list of women who had a breast cancer recorded in the EHR using the list of Read codes provided in Appendix 1. We will then restrict the list to women aged 18-80 years, who were registered with the same primary care practice for at least one year before the breast cancer diagnosis, and who are currently alive, registered with the same practice, and have passed the first anniversary of their cancer diagnosis. A list of Read codes for other cancers [[35](#_ENREF_35)] will be used to further exclude women who have had any other malignancy diagnosed before or after the breast cancer.

A random list of potentially eligible breast cancer survivors from each general practice will be selected. The number of women to be randomly selected from each practice will be calculated as the total number of women necessary for the study multiplied by the number of breast cancer survivors in the practice divided by the total number of potentially eligible breast cancer survivors in all practices.

The list of potentially eligible breast cancer survivors will be provided to the GP, and s/he will apply the following exclusion criteria:

1. The woman had a another cancer (not detected in the EHR), or has been treated for a non-invasive breast tumour;

b) The woman is considered unable to complete a self-administered questionnaire written in English for any reason.

The number of women excluded by the GP under each criterion will be recorded. Breast cancer survivors not excluded will be eligible for the study and invited to participate.

Women who did not have cancer

A list of Read codes [[35](#_ENREF_35)] will be used to exclude patients who have had cancer from the list of patients attending the same practices as the cancer survivors. In addition, patients who have not been registered continuously for the last two years with the practice and outside the age range 18-80 years will be excluded. Women still in the list are potentially eligible.

The number of women to be selected from each practice will be calculated as: total number of women without cancer necessary for the study times the number of women without cancer in the practice divided by the total number of women without cancer in all practices.

For each practice, we will then calculate the proportion of breast cancer survivors in the following age groups: 18-24, 25-34, 35-44, 45-54, 55-64, 65-74, 75-80. The final list of potentially eligible controls will be created by randomly selecting women with the same age distribution as of the breast cancer survivors of that same practice.

This list of potentially eligible controls will be sent to the GPs, and s/he will confirm that the women did not have a cancer and apply exclusion criteria a) and b).

Women not excluded will be considered eligible controls and invited to participate in the study.

## Data to be collected

## Health-related quality of life

Information on HRQoL will be collected using the Quality of Life in Adult Cancer Survivors Scale (QLACS) [[36](#_ENREF_36)]. The QLACS was developed to take into account the specific needs of long-term cancer survivors, including issues that continue after treatment, new issues that arise during the period post-cancer, late physical effects of the cancer treatments and positive aspects of surviving to cancer [[36](#_ENREF_36)]. It includes 47 items, divided in 7 generic and 5 cancer-specific domains (Appendix 2).

Breast cancer survivors will be asked to reply to all 47 items of the QLCAS (Appendix 3). Women who never had cancer will reply to the 28 items of the generic domains (Appendix 4).

## Anxiety and depression

Data on anxiety and depressive symptoms will be collected with the Hospital Anxiety and Depression Scale (HADS, 5) [[37](#_ENREF_37)]. This is a 14-item self-reported screening tool for anxiety and depressive symptoms in the past week. It contains two sub-scales, one for anxiety (HADS-A) and another for depression (HADS-D), with 7 items each [[37](#_ENREF_37)]. This scale has been validated for use in primary care [[38](#_ENREF_38)] and was used in primary care studies in the UK [[39-41](#_ENREF_39)].

## Clinical and socio-demographic data

Breast cancer survivors will be asked to provide information about the type of treatments received, the stage of their disease at diagnosis, the time since the last treatment (excluding long-term hormonal therapy), their menopausal status before and after the treatment, and how the cancer responded to the treatment (Appendix 6).

For all women, we will also collect data on potential confounders of the association between cancer history and mental health outcomes: education, ethnicity and social support (Appendix 7). Information on other potential confounders, such as co-morbidities or age at diagnosis will be obtained from the EHR.

## Data/statistical analysis

**Proportion of participation and exclusions**

The proportion of GPs who accept to participate in the study will be calculated for the whole of the UK, by country within the UK, and by region.

The proportion of patients considered by the GP as ineligible will be reported separately for breast cancer survivors and women who did not have cancer.

The proportion of breast cancer survivors who accept to participate in the study will be calculated, as well as the proportion of women who did not have cancer. The denominator will include all women in each group to whom questionnaires were sent, even though we expect a minor proportion of envelopes returned because the patient may have moved or died, or the address may not be correct.

## Objective 1: To describe cancer-specific measures of HRQoL in breast cancer survivors, and to explore the impact of demographic and clinical factors.

The QLACS includes 19 items for 5 cancer-specific domains of HRQoL (Appendix 2). Answers are provided on an ordinal Likert-type of scale, with values for individual items ranging from 1 to 7 [[36](#_ENREF_36)]. For each breast cancer survivor, we will group the items by domain and calculate the sum of the individual scores under each domain [[36](#_ENREF_36)]. All but one domain include 4 items; the “family distress” domain includes 3 items, and the sum of the individual scores will be rescaled to make the metric comparable with other domains. Values for each domain will range between 4 and 28. The range (minimum and maximum) scores will be reported for each domain, as well as the proportion of patients who score at the minimum and maximum values (floor and ceiling effects, respectively).

A mean or median score (depending on distribution) for each domain will be calculated from the individual-level sums of scores of the breast cancer survivors. Standard deviation will be calculated to quantify the dispersion of the data. The correlation coefficient among the mean scores of the domains will be reported.

A summary score for the cancer specific domains will be calculated by adding the mean/median scores of four domains (‘financial problems’, ‘distress-family’, ‘appearance’, and ‘distress-recurrence’); the mean/median score for ‘benefits from cancer’ is not included.

We will use linear regression models to estimate the association between the cancer-specific HRQoL domain scores and patients factors, such as stage at diagnosis or type of surgery. The dependent variable will be the sum of the individual items reported by each patient for that particular domain. The linear regression coefficients (β) from the regression models and the corresponding 95% confidence intervals will be reported.

## Objective 2: To compare generic measures of HRQoL between breast cancer survivors and women who did not have cancer, and to evaluate the impact of demographic and clinical variables.

The QLACS includes 28 items for 7 generic domains of HRQoL, with values for individual items ranging from 1 to 7 [[36](#_ENREF_36)] (Appendix 4).

The items will be grouped by domain (Appendix 2), and we will calculate, for each woman, the sum of the individual scores under each domain [[36](#_ENREF_36)]. For each group of participants (i.e. breast cancer survivors and women who did not have cancer), the range (minimum and maximum) of the scores will be reported for each domain, as well as the proportion of women who score at the minimum and maximum values of the domain.

A mean or median score, depending on the distribution of the data, will be obtained for each group of women, by calculating the mean/median of the sum of the scores for each woman in that group. The respective standard deviation will be reported.

A summary score for the generic domains will be calculated as the sum of the individual domain scores.

The student’s two-sample t-test, or a non-parametric alternative if needed (i.e. Mann-Whitney distribution free test), will be used to assess the evidence for a difference in the summary scores for each domain between the two groups.

Linear regression will be used to evaluate the impact of cancer diagnosis on the mean scores of HRQoL, adjusting for potential confounders. The role of socio-economic and clinical variables will be explored. The model fit and the linear regression coefficients (β) will be reported as well as the 95% confidence intervals.

## Objective 3: To compare the severity of depressive and anxiety symptoms in breast cancer survivors and in women who did not have cancer, and to assess the impact of clinical and demographic variables.

The Hospital Anxiety and Depression Scale contains two sub-scales, one for anxiety (HADS-A) and another for depression (HADS-D), with 7 items each [[37](#_ENREF_37)]. Each item is rated from 0 to 3 and the total score for each sub-scale ranges between 0 and 21; higher scores represent higher symptoms of depression or anxiety [[37](#_ENREF_37)].

To evaluate the severity of the depressive and anxiety symptoms in each group, the mean or median score, as appropriate, will be calculated for each sub-scale. The student’s t-test or the Mann-Whitney test will be used to compare the mean/median score of depressive and of anxiety symptoms between cancer survivors and women who did not have cancer.

To identify patients with clinically relevant symptoms of depression or anxiety, the authors of the scale propose the cut-off of 0-7 for non-cases, 8-10 for borderline cases and 11-21 for probable cases, in both subscales.

The proportion of patients falling into the three categories (non-case, borderline, probable case) will be estimated for breast cancer survivors and for controls.

A chi-squared test will be used to assess whether there is evidence of differences in the proportion of patients in these categories between the two groups. A test for trend will be used to evaluate if there are increasing changes over the categories in each group.

The participants will then be categorised as having or not having clinically relevant levels of depressive or anxiety symptoms (cut off >10). Logistic regression models will be used to estimate the association between breast cancer history and clinically relevant levels of anxiety, and breast cancer history and clinically relevant symptoms of depression. The impact of clinical and demographic variables will be explored in the regression models. Crude and adjusted odds ratios, and respective 95% confidence intervals, will be reported.

Alexander et al. [[42](#_ENREF_42)] evaluated the performance of the HADS as a screening test for major depressive disorder and anxiety in breast cancer survivors who were between 3 months and 2 years after main treatment conclusion (gold standard: non-patient Structured Clinical Interview for the Diagnostic and Statistical Manual of mental disorders (SCID)). Using the proposed cut-off of >10, the HADS-D had a sensitivity of 50% (95% confidence interval (95%CI): 27 to 73) and a specificity of 97% (95%CI: 93 to 99) [[42](#_ENREF_42)]. However, the HADS-A had a sensitivity of 71% (95%CI: 30 to 95) and specificity of 87% (95%CI: 81 to 91) [[42](#_ENREF_42)]. Even though the optimal cut-off for this population has not been established, a sensitivity of 50% may be too low to be acceptable in clinical practice, and therefore we will conduct a sensitivity analysis considering the cut-off of ≥8 to classify women as having clinically relevant symptoms of anxiety or depression.

## Objective 4: To compare the information reported by the patients for HRQoL, and for depressive and anxiety symptoms, with the information registered in the EHR for similar constructs

*HRQoL*

The QLACS includes seven generic domains of HRQoL (Appendix 4). Of these, five are particularly suitable for comparison with the data recorded in the EHR because women with distressing levels for these domains may have visited their GP to seek help: ‘negative feelings’, ‘cognitive problems’, ‘physical pain’, ‘sexual problems’ and ‘fatigue’. Read codes for the ‘social avoidance’ domain are also available, and therefore we included also this domain.

For each woman, we will calculate the mean score for each domain (mean values will range between 1 and 7). Then, we will consider as reporting important levels of distress all women with a mean score of ≥5 (corresponding to replies of frequently, very often or always to most questions) in the domains of negative feelings, cognitive problems, physical pain, sexual problems and fatigue. Two sensitivity analyses will be conducted: 1) using a lower cut-off of ≥3 (corresponding to replies of sometimes and as often as not, in addition to replies of frequently, very often or always to most questions); 2) considering as exposed to important levels of distress all women who replied ≥5 to at least one item in the domain.

To identify evidence of the corresponding outcomes in the EHR, we will produce a list of Read codes closely related to the QLACS items for each domain (table 1). This list of Read codes will be used to identify women (who have had and who did not have breast cancer) with these outcomes registered in their EHR in the previous year (or since the first anniversary of diagnosis, if a cancer was diagnosed at less than 2 years).

Table 1 Domains and respective items of the QLACS scale, and conditions related to each domain.

| **Domain** | **Items in the QLACS** | **Read codes* related to:** |
| --- | --- | --- |
|  |  |  |
| Negative feelings | 19 Bothered by mood swings;  7 Felt blue or depressed;  9 Worried about little things;  24 Felt anxious | Depression, anxiety |
| Cognitive problems | 3 Bothered by having a short attention span  4 Had trouble remembering things  2 Difficulty doing things requiring concentration  23 Bothered by forgetting what started to do | Mild cognitive impairment  Cognitive dysfunction |
| Physical pain | 13 Bothered by pain preventing activities  17 Mood disrupted by pain or its treatment  27 Pain interfered w/social activities  21 Had aches or pains | Pain reported as a symptom  Prescriptions of analgesics |
| Sexual problems | Sexual interest:  16 Lacked interest in sex  26 Avoided sexual activity  Sexual function  12 Dissatisfied w/sex life  10 Bothered by inability to function sexually | Sexual dysfunction  Hypoactive sexual disorder  Prescription of topical oestrogens |
| Fatigue | 11 Lacked energy to do things wanted to  14 Felt tired a lot  1 Had energy to do things wanted to do  5 Felt fatigued | Fatigue  Low energy |
| Social avoidance | 18 Avoided social gatherings  20 Avoided friends  25 Reluctant to meet new people  15 Reluctant to start new relationships | Social isolation  Social difficulties  Non aggressive unsocial conduct disorder |

* This will also be based on the systematic review of the Read codes used to identify mental health outcomes in primary care databases.

We will estimate the proportion of women who reported distressing levels for these domains, and the proportion of women who have a recording of a similar construct in the EHR, separately for breast cancer survivors and for women who did not have cancer.

To estimate how much inquiring the patient adds to the information registered in the EHR, we will calculate the probability of:

1. having information for a particular domain registered in the EHR, among women who reported distressing levels for that domain (sensitivity);
2. not having any information registered in the EHR for a particular domain among women who did not report distressing levels for that domain (specificity);
3. reporting distressing levels for a particular domain among women who had information for that domain registered in the EHR (positive predictive value);
4. not reporting distressing levels for a particular domain among women who did not have data for that domain registered in the EHR (negative predictive value).

All probabilities will be calculated separately for breast cancer survivors and for women who did not have cancer.

*Anxiety and depression*

The scores of the HADS-A and HADS-D will be used to classify women as having clinically relevant levels of anxiety and of depressive symptoms, respectively, using >10 as cut-off. The proportion of women scoring above this threshold will be calculated.

Women with a diagnosis of an anxiety and/or depressive disorder will be identified in the EHR through a list of Read codes. This list will be based on a systematic review of the literature to identify mental disorders in primary care databases. Women with a Read code for a depressive or anxiety disorder diagnosed in the last year will be considered depressed or anxious. A sensitivity analysis will include Read codes for symptoms of depression and/or anxiety, to account for the difficulties in the diagnosis of these conditions.

We will calculate, for each group of women and for each disorder, the probability of:

1. having a diagnosis of anxiety/depression registered in the EHR among women who scored above the threshold in the HADS scale (sensitivity);
2. not having a diagnosis of anxiety/depression registered in the EHR among women who did not score above the threshold in the HADS scale (specificity);
3. scoring above the threshold in the HADS scale among women who had a diagnosis of anxiety/depression recorded in the EHR (positive predictive value);
4. not scoring above the threshold in the HADS scale among women who did not have a diagnosis of anxiety/depression recorded in the EHR (negative predictive value).

## Plan for addressing missing data

We estimate that 5% of the women will have missing data for at least one item of the QLACS. This is a conservative estimate based on literature (the highest proportion of missing items was 3.2% [[43](#_ENREF_43)]). The HADS has been shown to have excellent acceptability [[37](#_ENREF_37)] and the proportion of missing items is usually small.

We will explore the pattern of missingness of the items by demographic and clinical variables. For that purpose, a variable will be created to denote records with incomplete information and we will explore the association between this variable and clinical and demographic variables. If the missingness can be explained by the other variables in the dataset, we will consider that it is missing at random, and specify a multiple imputation model to better represent the distribution from which the missing data came.

## Sample size

We estimate that a sample of 260 breast cancer survivors and 260 women who did not have cancer are required to detect differences of the size reported in the literature. As participation rate in this type of studies has been low (approximately 20%), we believe that 1,400 women in each group need to be invited.

*HRQoL*

Table 2 provides details of the sample size calculation for the comparison of the summary scores of HRQoL, and of the mean scores of the generic domains of HRQoL, between breast cancer survivors and women who did not have cancer.

Table 2 Estimated sample size to compare the mean values of HRQoL in breast cancer survivors and women who did not have cancer.

|  | HRQoL mean score breast cancer survivors (SD) | HRQoL mean score normative data (SD) | Sample size  per group ^†^ | Adjusted* sample size  per group |
| --- | --- | --- | --- | --- |
|  |  |  |  |  |
| Summary score | 68.5 (22.7) ^1^ | 60.9 (21.5) | 133 | 800 |
| Summary score | 70.5 (26.6) ^2^ | 60.9 (21.5) | 100 | 600 |
| Summary score | 75.5 (26.3) ^2^ | 60.9 (21.5) | 43 | 350 |
|  |  |  |  |  |
| **Generic domains** |  |  |  |  |
| Negative feelings | 9.7 (3.8) | 7.1 (3.5) | 31 | 300 |
| Positive feelings | 22.1 (4.7) | 20.3 (6.3) | 85 | 550 |
| Cognitive problems | 9.8 (5.0) | 8.3 (2.7) | 113 | 700 |
| Sexual problems | 11.8 (6.8) | 9.0 (3.4) | 58 | 400 |
| Physical pain | 9.7 (6.1) | 7.8 (4.8) | 131 | 800 |
| Fatigue | 11.8 (5.4) | 10.3 (4.6) | **176** | **1,000** |
| Social avoidance | 8.2 (4.3) | 6.9 (2.8) | 123 | 750 |
|  |  |  |  |  |

† Assuming an alpha of 0.05 and power of 80%.

* Calculated as the estimated sample size rounded upwards to the next 10 subjects (to take into account the uncertainty of the estimation process) divided by 0.2 (the estimated proportion of participation), and added a 100 patients to account for other variables to be studied.

^1^ Women diagnosed with breast cancer at 18-24 months [[44](#_ENREF_44)].

^2^ Women diagnosed with breast cancer at 5 years of more [[36](#_ENREF_36), [43](#_ENREF_43)].

The summary mean scores for the generic domains of the QLACS among breast cancer survivors were obtained from the literature [[36](#_ENREF_36), [43](#_ENREF_43), [44](#_ENREF_44)]. The mean/median scores of the generic domains among women who did not have cancer have not been reported. However, in a study involving long-term survivors of breast, bladder, head and neck, gynaecologic, prostate and colorectal cancer [[36](#_ENREF_36)], patients with colorectal cancer ranked the lowest summary score (indicating better HRQoL) for the generic domains of HRQoL (mean 60.9, SD=21.5). We used this score as a conservative estimate of the summary score of HRQoL in the general population, assuming that women who never have had cancer will not have worse HRQoL than the cancer patients who experience the best HRQoL. The same assumption was applied to estimate the sample size for the specific domains of HRQoL.

*Anxiety and depression*

Table 3 provides sample size estimates for the comparison of the mean scores of the two subscales of the HADS. As shown in the table, one study found a difference in mean HADS-Depression scores of just 0.6; to detect such a small difference would require 447 women per group, which would be beyond available resources. However, another study has calculated that differences of less than 1.4 in mean HADS-depression scores are not clinically important [[45](#_ENREF_45)], and only around 75 patients per group would be required to detect differences above this level. For anxiety we would require 253 women per group to detect the minimum previously observed differences on the HADS scale.

Table 3 Estimated sample size to compare the mean scores of anxiety and depression between breast cancer survivors and women from the general population.

|  | Mean score breast cancer survivors (SD) | Mean score normative data (SD) | Sample size  per group ^†^ | Adjusted* sample size  per group |
| --- | --- | --- | --- | --- |
|  |  |  |  |  |
| HADS-Anxiety | 6.3 (2.8) [[46](#_ENREF_46)] | 4.8 (3.7) [[46](#_ENREF_46)] | 76 | 500 |
| HADS-Anxiety | 7.8 (3.0) [[47](#_ENREF_47)] | 7.1 (2.6) [[47](#_ENREF_47)] | **253** | **1,400** |
|  |  |  |  |  |
| HADS-Depression | 3.1 (3.3) [[46](#_ENREF_46)] | 3.7 (3.1) [[46](#_ENREF_46)] | (447) | (2,350) |
| HADS-Depression | 4.6 (3.3) [[47](#_ENREF_47)] | 3.2 (2.7) [[47](#_ENREF_47)] | 73 | 500 |
|  |  |  |  |  |

* Calculated as the estimated sample size rounded upwards to the next 10 subjects (to take into account the uncertainty of the estimation process) divided by 0.2 (the estimated proportion of participation), and added a 100 patients to account for other variables to be studied.

Table 4 provides estimates of the number of women necessary to compare the prevalences of anxiety and depression, as determined by the cut-offs of the HADS [[48](#_ENREF_48)].

Table 4 Estimated sample size to compare the prevalences of anxiety and depression between breast cancer survivors and women from the general population.

| Outcome | α | β | % of outcome in unexposed group [ref] | Estimated risk ratio  [ref] | Sample size  per group ^†^ | Adjusted* sample size  per group |
| --- | --- | --- | --- | --- | --- | --- |
|  |  |  |  |  |  |  |
| Anxiety | 0.05 | 0.20 | 36.5 [[48](#_ENREF_48)] | 1.44 [[48](#_ENREF_48)] | 150 | 850 |
|  |  |  |  |  |  |  |
| Depression | 0.05 | 0.20 | 12.9 [[48](#_ENREF_48)] | 1.21 [[48](#_ENREF_48)] | **2,614** | **13,170** |
|  |  |  |  |  |  |  |

* Calculated as the estimate sample size rounded upwards to the next 10 subjects (to take into account the uncertainty of the estimation process) divided by 0.2 (the estimated proportion of participation), and added a 100 patients to account for other variables to be studied.

According to the calculations, over 13,000 breast cancer survivors and 13,000 women who did not have cancer would be needed to compare the prevalence of depression between the two groups of women. Recruiting more than 1,500 women for this study is not feasible, and therefore we chose the sample size necessary to compare the mean scores of anxiety and depression between the two groups (n=1,400 in each group, as outlined above and in Table 3).

## Feasibility counts

A total of 43,704 women with breast cancer, and who were at least one year post-diagnosis, were identified in the July 2015 cut of the CPRD primary care database. Of these, 21,564 women had acceptable records from practices contributing with ‘up to standard’ data. A total of 8,763 women were still registered in practices that contributed with data to CPRD during the year of 2016, of which 7,498 (86%) were aged between 18 and 80 years old. Table 5 describes the distribution of the patients by region within England.

Table 5 Number of women and general practices with active records (as per June 2016) in the CPRD primary care database, by region.

| Region code | Region label | No. of  practices | No. of  patients |
| --- | --- | --- | --- |
|  |  |  |  |
| 1 | North East | 3 | 91 |
| 2 | North West | 28 | 1,139 |
| 3 | Yorkshire & The Humber | 3 | 160 |
| 4 | East Midlands | 0 | 0 |
| 5 | West Midlands | 18 | 763 |
| 6 | East of England | 10 | 557 |
| 7 | South West | 17 | 658 |
| 8 | South Central | 27 | 1,304 |
| 9 | London | 35 | 1,007 |
| 10 | South East Coast | 47 | 1,819 |
|  |  |  |  |
|  | Total | 188 | 7,437 |

The estimated sample size (1,400) corresponds to 19% of the women potentially eligible for the study.

## Pilot study

We will invite all GPs working in practices contributing with ‘up to standard’ data to CPRD at the time of recruitment to participate in the study.

Packages containing paper questionnaires will be sent to 140 breast cancer survivors and 140 women who did not have cancer (10% of those to be invited), randomly selected from the list of patients attending the first practices to sign up for the study. The pilot phase will run for 1 month. After that time, we will estimate:

1. the proportion of participation in each group;
2. the age distribution of the participants in each group;
3. the number of questionnaires with missing items.

Sample size calculations will be revised, if necessary. Afterwards, paper questionnaires will be sent out to the remainder of women to be invited, up to the estimated sample size.

## Limitations of the study design, data sources and analytical methods

We will use the CPRD primary care database to classify women as exposed or not to breast cancer. CPRD has been shown to capture more than 90% of the cancer diagnoses registered in the cancer registries [[49](#_ENREF_49)]. This is considered acceptable for this project, even though a small proportion of the women may be incorrectly classified as unexposed. We will request that the GP revises the list of patients to exclude potentially misclassified cases.

We expect a substantial proportion of patients to decline to participate in the study, as shown by the proportion of participation in previous studies. Selection bias may occur if the patients who accept to participate in the study differ systematically from those who do not. We will compare the demographic characteristics of the women who participate in the study with the broad characteristics of the women who had breast cancer in the CPRD primary care database. Also, we assumed a similar participation rate by age-group between women with breast with and without cancer. We will compare the age-distribution of the final samples and take age into account in multivariate analyses if necessary.

Women who are unable to complete a self-administered questionnaire due to advanced disease (e.g. terminally ill, patients with dementia or severe mental illnesses) will be excluded from the study. Therefore, the generalizability of our results will be limited women with a relatively good cognitive function.

The QLACS was validated in the United States but not in the UK population of cancer survivors. However, no translation is required and the entire scale will be applied, which makes unlikely the occurrence of substantial bias.

This study will have limited power to detect a strong association between having had a breast cancer and depression as defined by the cut-offs of the HADS scale. Our primary outcome will be the difference of the mean scores of each sub-scale, for which this study will have enough power.

## Patient or user group involvement

Two women who never had cancer revised the invitation letter, participant information sheets and questionnaires for women in the non-cancer comparison group.

Breast cancer survivors identified through the Independent Cancer Patients’ Voice (a patient advocate group and charity) revised the materials for breast cancer survivors. Comments from each group were incorporated into the study materials.

We will also ask selected members of the public and breast cancer survivors to comment on the report produced to share the study results prior to making these available.

## Plans for disseminating and communicating study results, including the presence or absence of any restrictions on the extent and timing of publication

We plan to disseminate the results with the publication of an article in a peer-reviewed scientific journal. We will also present preliminary finding at scientific meetings.

To share the results with the general public, we will make the study results publicly available online. We will create a study’s webpage on the website of the London School of Hygiene & Tropical Medicine. The website address for this webpage will be included in the participant information packs. A summary of findings from the study will be posted on the study webpage in due course. Anyone visiting this webpage (whether a participant, invitee, general practitioner or any interested member of the public) will be able to provide a contact email address through the webpage to subscribe for updates. The study researchers will use these contact email addresses for the sole purpose of letting interested parties know about updates to the study webpage.

## Timetable


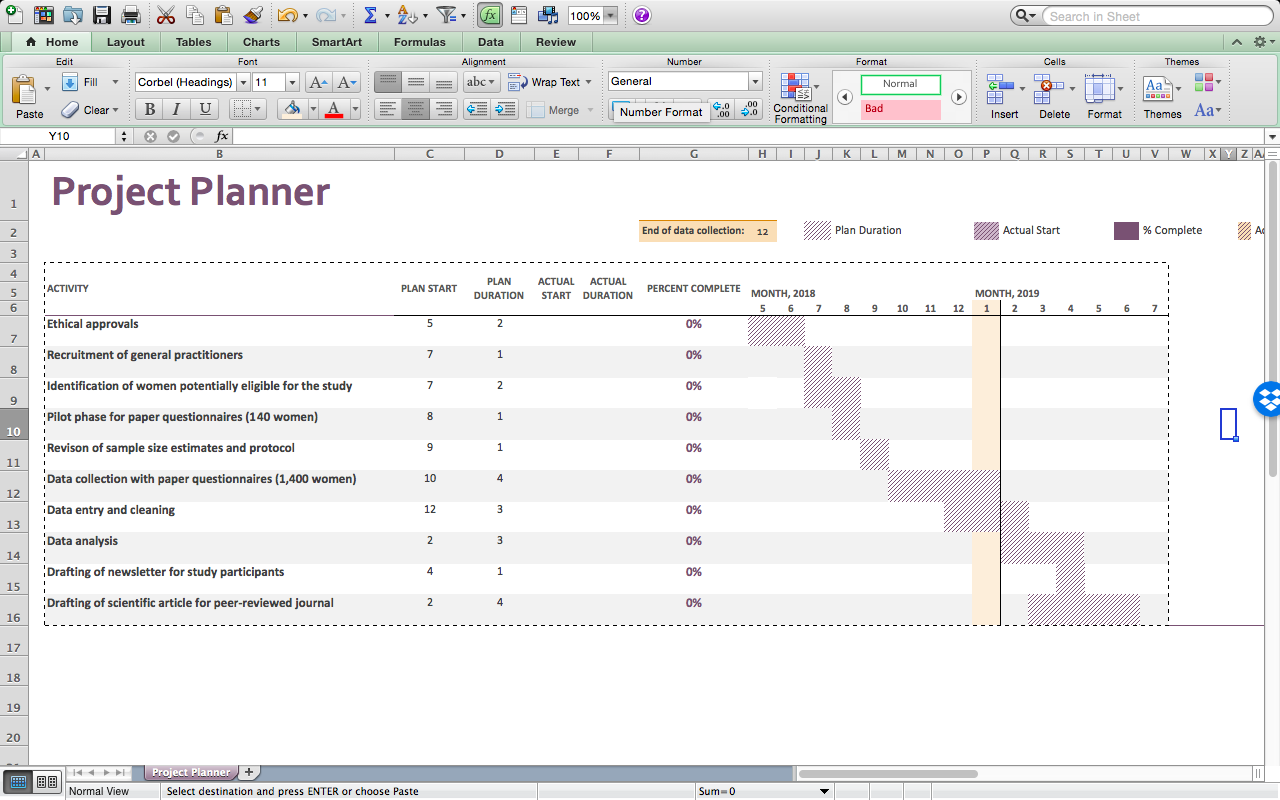

## References

1. Forman D, et al., eds. *Cancer Incidence in Five Continents, Vol. X*. IARC Scientific Publication No. 164. 2014, International Agency for Research on Cancer: Lyon.

2. Allemani, C., et al., *Global surveillance of cancer survival 1995-2009: analysis of individual data for 25,676,887 patients from 279 population-based registries in 67 countries (CONCORD-2).* Lancet, 2015. **385**(9972): p. 977-1010.

3. Ferlay, J., et al., *GLOBOCAN 2012 v1.0, Cancer Incidence and Mortality Worldwide: IARC CancerBase No. 11 [Internet].* 2013: Lyon, France: International Agency for Research on Cancer.

4. Maddams, J., M. Utley, and H. Moller, *Projections of cancer prevalence in the United Kingdom, 2010-2040.* Br J Cancer, 2012. **107**(7): p. 1195-202.

5. Weisman, A.D. and J.W. Worden, *The existential plight in cancer: significance of the first 100 days.* Int J Psychiatry Med, 1976. **7**(1): p. 1-15.

6. Schubart, J.R., et al., *Screening for psychological distress in surgical breast cancer patients.* Ann Surg Oncol, 2014. **21**(10): p. 3348-53.

7. Al-Azri, M., H. Al-Awisi, and M. Al-Moundhri, *Coping with a diagnosis of breast cancer-literature review and implications for developing countries.* Breast J, 2009. **15**(6): p. 615-22.

8. Senkus, E., et al., *Primary breast cancer: ESMO Clinical Practice Guidelines for diagnosis, treatment and follow-up.* Ann Oncol, 2015. **26 Suppl 5**: p. v8-30.

9. Caffo, O., et al., *Pain and quality of life after surgery for breast cancer.* Breast Cancer Res Treat, 2003. **80**(1): p. 39-48.

10. Bower, J.E., et al., *Fatigue in breast cancer survivors: occurrence, correlates, and impact on quality of life.* J Clin Oncol, 2000. **18**(4): p. 743-53.

11. Tsai, R.J., et al., *The risk of developing arm lymphedema among breast cancer survivors: a meta-analysis of treatment factors.* Ann Surg Oncol, 2009. **16**(7): p. 1959-72.

12. Andersen, K.G. and H. Kehlet, *Persistent pain after breast cancer treatment: a critical review of risk factors and strategies for prevention.* J Pain, 2011. **12**(7): p. 725-46.

13. Helms, R.L., E.L. O'Hea, and M. Corso, *Body image issues in women with breast cancer.* Psychol Health Med, 2008. **13**(3): p. 313-25.

14. Ahles, T.A., et al., *Neuropsychologic impact of standard-dose systemic chemotherapy in long-term survivors of breast cancer and lymphoma.* J Clin Oncol, 2002. **20**(2): p. 485-93.

15. Jim, H.S., et al., *Meta-analysis of cognitive functioning in breast cancer survivors previously treated with standard-dose chemotherapy.* J Clin Oncol, 2012. **30**(29): p. 3578-87.

16. Howard-Anderson, J., et al., *Quality of life, fertility concerns, and behavioral health outcomes in younger breast cancer survivors: a systematic review.* J Natl Cancer Inst, 2012. **104**(5): p. 386-405.

17. Thewes, B., et al., *Fertility- and menopause-related information needs of younger women with a diagnosis of early breast cancer.* J Clin Oncol, 2005. **23**(22): p. 5155-65.

18. Kim, Y., et al., *Quality of life of couples dealing with cancer: dyadic and individual adjustment among breast and prostate cancer survivors and their spousal caregivers.* Ann Behav Med, 2008. **35**(2): p. 230-8.

19. Cohen, M. and S. Pollack, *Mothers with breast cancer and their adult daughters: the relationship between mothers' reaction to breast cancer and their daughters' emotional and neuroimmune status.* Psychosom Med, 2005. **67**(1): p. 64-71.

20. Raveis, V.H. and S. Pretter, *Existential plight of adult daughters following their mother's breast cancer diagnosis.* Psychooncology, 2005. **14**(1): p. 49-60.

21. Hoving, J.L., M.L. Broekhuizen, and M.H. Frings-Dresen, *Return to work of breast cancer survivors: a systematic review of intervention studies.* BMC Cancer, 2009. **9**: p. 117.

22. Koch-Gallenkamp, L., et al., *Fear of Recurrence in Long-Term Cancer Survivors-Do Cancer Type, Sex, Time Since Diagnosis, and Social Support Matter?* Health Psychol, 2016.

23. Tiedtke, C., et al., *Survived but feeling vulnerable and insecure: a qualitative study of the mental preparation for RTW after breast cancer treatment.* BMC Public Health, 2012. **12**: p. 538.

24. Boykoff, N., M. Moieni, and S.K. Subramanian, *Confronting chemobrain: an in-depth look at survivors' reports of impact on work, social networks, and health care response.* J Cancer Surviv, 2009. **3**(4): p. 223-32.

25. Hunter, M.S., et al., *Menopausal symptoms following breast cancer treatment: a qualitative investigation of cognitive and behavioural responses.* Maturitas, 2009. **63**(4): p. 336-40.

26. Patrick D, Guyatt GH, and Acquadro C, *Chapter 17: Patient-reported outcomes. In: Higgins JPT, Green S (editors), Cochrane Handbook for Systematic Reviews of Interventions Version 5.1.0 (updated March 2011). The Cochrane Collaboration. Available from* <http://www.cochrane-handbook.org>. 2011.

27. Khan, N.F., et al., *Consulting and prescribing behaviour for anxiety and depression in long-term survivors of cancer in the UK.* Eur J Cancer, 2010. **46**(18): p. 3339-44.

28. Department of Health – Quality Health, *Quality of life of cancer survivors in England: Report on a pilot survey using Patient Reported Outcome Measures (PROMS)*. 2012.

29. Herrett, E., et al., *Data Resource Profile: Clinical Practice Research Datalink (CPRD).* Int J Epidemiol, 2015. **44**(3): p. 827-36.

30. National Institute for Health and Care Excellence (NICE), *Depression in adults with a chronic physical health problem: recognition and management (CG91)*. 2009.

31. National Institute for Health and Care Excellence (NICE), *Generalised anxiety disorder and panic disorder in adults: management (CG113)*. 2011.

32. Johns, C., et al., *Informing hot flash treatment decisions for breast cancer survivors: a systematic review of randomized trials comparing active interventions.* Breast Cancer Res Treat, 2016. **156**(3): p. 415-426.

33. Cella, D. and L.J. Fallowfield, *Recognition and management of treatment-related side effects for breast cancer patients receiving adjuvant endocrine therapy.* Breast Cancer Res Treat, 2008. **107**(2): p. 167-80.

34. Suppli, N.P., et al., *Increased risk for depression after breast cancer: a nationwide population-based cohort study of associated factors in Denmark, 1998-2011.* J Clin Oncol, 2014. **32**(34): p. 3831-9.

35. Bhaskaran, K., et al., *Body-mass index and risk of 22 specific cancers: a population-based cohort study of 5.24 million UK adults.* Lancet, 2014. **384**(9945): p. 755-65.

36. Avis, N.E., et al., *Assessing quality of life in adult cancer survivors (QLACS).* Qual Life Res, 2005. **14**(4): p. 1007-23.

37. Zigmond, A.S. and R.P. Snaith, *The hospital anxiety and depression scale.* Acta Psychiatr Scand, 1983. **67**(6): p. 361-70.

38. Wilkinson, M.J. and P. Barczak, *Psychiatric screening in general practice: comparison of the general health questionnaire and the hospital anxiety depression scale.* J R Coll Gen Pract, 1988. **38**(312): p. 311-3.

39. Thompson, C., et al., *Dimensional perspective on the recognition of depressive symptoms in primary care: The Hampshire Depression Project 3.* Br J Psychiatry, 2001. **179**: p. 317-23.

40. Ostler, K., et al., *Influence of socio-economic deprivation on the prevalence and outcome of depression in primary care: the Hampshire Depression Project.* Br J Psychiatry, 2001. **178**(1): p. 12-7.

41. Mallen, C.D. and G. Peat, *Screening older people with musculoskeletal pain for depressive symptoms in primary care.* Br J Gen Pract, 2008. **58**(555): p. 688-93.

42. Alexander, S., C. Palmer, and P.C. Stone, *Evaluation of screening instruments for depression and anxiety in breast cancer survivors.* Breast Cancer Res Treat, 2010. **122**(2): p. 573-8.

43. Avis, N.E., E. Ip, and K.L. Foley, *Evaluation of the Quality of Life in Adult Cancer Survivors (QLACS) scale for long-term cancer survivors in a sample of breast cancer survivors.* Health Qual Life Outcomes, 2006. **4**: p. 92.

44. Sohl, S.J., B. Levine, and N.E. Avis, *Evaluation of the Quality of Life in Adult Cancer Survivors (QLACS) scale for early post-treatment breast cancer survivors.* Qual Life Res, 2015. **24**(1): p. 205-12.

45. Puhan, M.A., et al., *The minimal important difference of the hospital anxiety and depression scale in patients with chronic obstructive pulmonary disease.* Health Qual Life Outcomes, 2008. **6**: p. 46.

46. Dahl, A.A., et al., *Arm/shoulder problems and insomnia symptoms in breast cancer survivors: cross-sectional, controlled and longitudinal observations.* Sleep Med, 2011. **12**(6): p. 584-90.

47. Calvio, L., et al., *Measures of cognitive function and work in occupationally active breast cancer survivors.* J Occup Environ Med, 2010. **52**(2): p. 219-27.

48. Boehmer, U., A. Ozonoff, and J. Potter, *Sexual Minority Women's Health Behaviors and Outcomes After Breast Cancer.* LGBT Health, 2015. **2**(3): p. 221-7.

49. Boggon, R., et al., *Cancer recording and mortality in the General Practice Research Database and linked cancer registries.* Pharmacoepidemiol Drug Saf, 2013. **22**(2): p. 168-75.

50. Ranopa, M., et al., *The identification of incident cancers in UK primary care databases: a systematic review.* Pharmacoepidemiol Drug Saf, 2015. **24**(1): p. 11-8.

## Appendices

### Appendix 1. List of read codes to identify breast cancer patients [[50](#_ENREF_50)].

| **Read Code** | **Description** |
| --- | --- |
|  |  |
| B34..11 | CA FEMALE BREAST |
| B36..00 | LOCAL RECURRENCE OF MALIGNANT TUMOUR OF BREAST |
| B340100 | MALIGNANT NEOPLASM OF AREOLA OF FEMALE BREAST |
| B346.00 | MALIGNANT NEOPLASM OF AXILLARY TAIL OF FEMALE BREAST |
| B341.00 | MALIGNANT NEOPLASM OF CENTRAL PART OF FEMALE BREAST |
| B34y000 | MALIGNANT NEOPLASM OF ECTOPIC SITE OF FEMALE BREAST |
| B34..00 | MALIGNANT NEOPLASM OF FEMALE BREAST |
| B34z.00 | MALIGNANT NEOPLASM OF FEMALE BREAST NOS |
| B343.00 | MALIGNANT NEOPLASM OF LOWER-INNER QUADRANT OF FEMALE BREAST |
| B345.00 | MALIGNANT NEOPLASM OF LOWER-OUTER QUADRANT OF FEMALE BREAST |
| B340.00 | MALIGNANT NEOPLASM OF NIPPLE AND AREOLA OF FEMALE BREAST |
| B340000 | MALIGNANT NEOPLASM OF NIPPLE OF FEMALE BREAST |
| B340z00 | MALIGNANT NEOPLASM OF NIPPLE OR AREOLA OF FEMALE BREAST NOS |
| B34y.00 | MALIGNANT NEOPLASM OF OTHER SITE OF FEMALE BREAST |
| B34yz00 | MALIGNANT NEOPLASM OF OTHER SITE OF FEMALE BREAST NOS |
| B342.00 | MALIGNANT NEOPLASM OF UPPER-INNER QUADRANT OF FEMALE BREAST |
| B344.00 | MALIGNANT NEOPLASM OF UPPER-OUTER QUADRANT OF FEMALE BREAST |
| B347.00 | MALIGNANT NEOPLASM, OVERLAPPING LESION OF BREAST |
| BB93.00 | [M]COMEDOCARCINOMA NOS |
| BBM9.00 | [M]CYSTOSARCOMA PHYLLODES, MALIGNANT |
| BB91100 | [M]INFILTRATING DUCT AND LOBULAR CARCINOMA |
| BB91.00 | [M]INFILTRATING DUCT CARCINOMA |
| BB9G.00 | [M]INFILTRATING DUCTULAR CARCINOMA |
| BB9H.00 | [M]INFLAMMATORY CARCINOMA |
| BB91000 | [M]INTRADUCTAL PAPILLARY ADENOCARCINOMA WITH INVASION |
| BB94.00 | [M]JUVENILE BREAST CARCINOMA |
| BB9F.00 | [M]LOBULAR CARCINOMA NOS |
| BB9D.00 | [M]MEDULLARY CARCINOMA WITH LYMPHOID STROMA |
| BB9K.00 | [M]PAGET'S DISEASE AND INFILTRATING BREAST DUCT CARCINOMA |
| BB9K000 | [M]PAGET'S DISEASE AND INTRADUCTAL CARCINOMA OF BREAST |
| BB9J.11 | [M]PAGET'S DISEASE, BREAST |
| BB9J.00 | [M]PAGET'S DISEASE, MAMMARY |
| BB94.11 | [M]SECRETORY BREAST CARCINOMA |
| Byu6.00 | [X]MALIGNANT NEOPLASM OF BREAST |

### Appendix 2. Items of the Quality of Life in Adult Cancer Survivors Scale (QLACS) grouped by domain.

| **Domain** | | | | **Item of the Quality of Life in Adult Cancer Survivors scale [**[**36**](#_ENREF_36)**]** |
| --- | --- | --- | --- | --- |
|  | | | | |
| **Generic** | | | | |
|  | *Negative feelings* | | | |
|  |  | 19 Bothered by mood swings | | |
|  |  | 7 Felt blue or depressed | | |
|  |  | 9 Worried about little things | | |
|  |  | 24 Felt anxious | | |
|  |  |  | | |
|  | *Positive feelings* | | | |
|  |  | 8 Enjoyed life | | |
|  |  | 28 Content with life | | |
|  |  | 6 Felt happy | | |
|  |  | 22 Had a positive outlook on life | | |
|  |  |  | | |
|  | *Cognitive problems* | | | |
|  |  | 3 Bothered by having a short attention span | | |
|  |  | 4 Had trouble remembering things | | |
|  |  | 2 Difficulty doing things requiring concentration | | |
|  |  | 23 Bothered by forgetting what started to do | | |
|  |  |  | | |
|  | *Pain* | | | |
|  |  | 13 Bothered by pain preventing activities | | |
|  |  | 17 Mood disrupted by pain or its treatment | | |
|  |  | 27 Pain interfered w/social activities | | |
|  |  | 21 Had aches or pains | | |
|  |  |  | | |
|  | *Sexual interest* | | | |
|  |  | 16 Lacked interest in sex | | |
|  |  | 26 Avoided sexual activity | | |
|  |  |  | | |
|  | *Energy/fatigue* | | | |
|  |  | 11 Lacked energy to do things wanted to | | |
|  |  | 14 Felt tired a lot | | |
|  |  | 1 Had energy to do things wanted to do | | |
|  |  | 5 Felt fatigued | | |
|  |  |  | | |
|  | *Sexual function* | | | |
|  |  | 12 Dissatisfied w/sex life | | |
|  |  | 10 Bothered by inability to function sexually | | |
|  |  | | | |
|  | *Social avoidance* | | | |
|  |  | 18 Avoided social gatherings | | |
|  |  | 20 Avoided friends | | |
|  |  | 25 Reluctant to meet new people | | |
|  |  | 15 Reluctant to start new relationships | | |
|  |  |  | | |
|  |  |  | | |
|  |  |  | | |
|  |  |  | | |
|  |  |  | | |
|  |  |  | | |
| **Cancer-specific** | | | | |
|  | | | | |
|  | *Financial problems* | | | |
|  |  | | 43 Had money problems from cancer | |
|  |  | | 45 Financial problems from loss of income due to cancer | |
|  |  | | 30 Financial problems from cost of cancer surgery or tx | |
|  |  | | 37 Problems with insurance because of cancer | |
|  |  | |  | |
|  | *Benefits* | | | |
|  |  | | 40 Cancer helped recognize what important in life | |
|  |  | | 41 Better able to deal w/stress because of cancer | |
|  |  | | 32 Cancer helped cope better w/problems | |
|  |  | | 29 Appreciated life more because of cancer | |
|  |  | |  | |
|  | *Distress-family* | | | |
|  |  | | 34 Worried whether family had cancer causing genes | |
|  |  | | 31 Worried family members were at risk for cancer | |
|  |  | | 42 Worried family should have genetic tests - cancer | |
|  |  | |  | |
|  | *Appearance* | | | |
|  |  | | 35 Felt unattractive b/c of cancer or its treatment | |
|  |  | | 33 Self-conscious about appearance because of cancer | |
|  |  | | 44 Felt treated differently b/c of changes in appearance | |
|  |  | | 38 Bothered by hair loss from cancer treatments | |
|  |  | | | |
|  | *Distress-recurrence* | | | |
|  |  | | 39 Worried about cancer coming back | |
|  |  | | 46 When felt pain, worried it was cancer again | |
|  |  | | 36 Worried about dying from cancer | |
|  |  | | 47 Preoccupied with concerns about cancer | |

### Appendix 3. Quality of Life in Adult Cancer Survivors Scale (QLACS) [[36](#_ENREF_36)]

We would like to ask you about some things that can affect the quality of people’s lives. Some of these questions may sound similar, but please be sure to answer each one. Below is a scale ranging from never to always. Please indicate how often each of these statements has been true for you in the past four weeks. [Select one answer for each question]

|  | | | **Never** | **Seldom** | **Sometimes** | **About as often as not** | **Frequently** | **Very often** | **Always** | |
| --- | --- | --- | --- | --- | --- | --- | --- | --- | --- | --- |
| **In the past 4 weeks…** | | | | | | | | | | |
| 1 | You had the energy to do the things you wanted to do. | | 1 | 2 | 3 | 4 | 5 | 6 | | 7 |
| 2 | You had difficulty doing activities that require concentrating. | | 1 | 2 | 3 | 4 | 5 | 6 | | 7 |
| 3 | You were bothered by having a short attention span. | | 1 | 2 | 3 | 4 | 5 | 6 | | 7 |
| 4 | You had trouble remembering things. | | 1 | 2 | 3 | 4 | 5 | 6 | | 7 |
| 5 | You felt fatigued. | | 1 | 2 | 3 | 4 | 5 | 6 | | 7 |
| 6 | You felt happy. | | 1 | 2 | 3 | 4 | 5 | 6 | | 7 |
| 7 | You felt blue or depressed. | | 1 | 2 | 3 | 4 | 5 | 6 | | 7 |
| 8 | You enjoyed life. | | 1 | 2 | 3 | 4 | 5 | 6 | | 7 |
| 9 | You worried about little things. | | 1 | 2 | 3 | 4 | 5 | 6 | | 7 |
| 10 | You were bothered by being unable to function sexually. | | 1 | 2 | 3 | 4 | 5 | 6 | | 7 |
| 11 | You didn’t have energy to do the things you wanted to do. | | 1 | 2 | 3 | 4 | 5 | 6 | | 7 |
| 12 | You were dissatisfied with your sex life. | | 1 | 2 | 3 | 4 | 5 | 6 | | 7 |
| 13 | You were bothered by pain that kept you from doing the things you wanted to do. | | 1 | 2 | 3 | 4 | 5 | 6 | | 7 |
| 14 | You felt tired a lot. | | 1 | 2 | 3 | 4 | 5 | 6 | | 7 |
| 15 | You were reluctant to start new relationships. | | 1 | 2 | 3 | 4 | 5 | 6 | | 7 |
| 16 | You lacked interest in sex. | | 1 | 2 | 3 | 4 | 5 | 6 | | 7 |
| 17 | Your mood was disrupted by pain or its treatment. | | 1 | 2 | 3 | 4 | 5 | 6 | | 7 |
| 18 | You avoided social gatherings. | | 1 | 2 | 3 | 4 | 5 | 6 | | 7 |
| 19 | You were bothered by mood swings. | | 1 | 2 | 3 | 4 | 5 | 6 | | 7 |
| 20 | You avoided your friends. | | 1 | 2 | 3 | 4 | 5 | 6 | | 7 |
| 21 | You had aches or pains. | | 1 | 2 | 3 | 4 | 5 | 6 | | 7 |
| 22 | You had a positive outlook on life. | | 1 | 2 | 3 | 4 | 5 | 6 | | 7 |
| 23 | You were bothered by forgetting what you started to do. | | 1 | 2 | 3 | 4 | 5 | 6 | | 7 |
| 24 | You felt anxious. | | 1 | 2 | 3 | 4 | 5 | 6 | | 7 |
| 25 | You were reluctant to meet new people. | | 1 | 2 | 3 | 4 | 5 | 6 | | 7 |
| 26 | You avoided sexual activity. | | 1 | 2 | 3 | 4 | 5 | 6 | | 7 |
| 27 | Pain or its treatment interfered with your social activities. | | 1 | 2 | 3 | 4 | 5 | 6 | | 7 |
| 28 | You were content with your life. | | 1 | 2 | 3 | 4 | 5 | 6 | | 7 |
|  |  | |  |  |  |  |  |  | |  |
| **The next set of questions asks specifically about the effects of your cancer or its treatment. Again, for each statement, indicate how often each of these statements has been true for you in the past four weeks.** | | | | | | | | | | |
| 29 | | You appreciated life more because of having had cancer. | 1 | 2 | 3 | 4 | 5 | 6 | | 7 |
| 30 | | You had financial problems because of the cost of cancer surgery or treatment. | 1 | 2 | 3 | 4 | 5 | 6 | | 7 |
| 31 | | You worried that your family members were at risk of getting cancer. | 1 | 2 | 3 | 4 | 5 | 6 | | 7 |
| 32 | | You realized that having had cancer helps you cope better with problems now. | 1 | 2 | 3 | 4 | 5 | 6 | | 7 |
| 33 | | You were self-conscious about the way you look because of your cancer or its treatment. | 1 | 2 | 3 | 4 | 5 | 6 | | 7 |
| 34 | | You worried about whether your family members might have cancer-causing genes. | 1 | 2 | 3 | 4 | 5 | 6 | | 7 |
| 35 | | You felt unattractive because of your cancer or its treatment. | 1 | 2 | 3 | 4 | 5 | 6 | | 7 |
| 36 | | You worried about dying from cancer. | 1 | 2 | 3 | 4 | 5 | 6 | | 7 |
| 37 | | You had problems with insurance because of cancer. | 1 | 2 | 3 | 4 | 5 | 6 | | 7 |
| 38 | | You were bothered by hair loss from cancer treatment. | 1 | 2 | 3 | 4 | 5 | 6 | | 7 |
| 39 | | You worried about cancer coming back. | 1 | 2 | 3 | 4 | 5 | 6 | | 7 |
| 40 | | You felt that cancer helped you to recognize what is important in life. | 1 | 2 | 3 | 4 | 5 | 6 | | 7 |
| 41 | | You felt better able to deal with stress because of having had cancer. | 1 | 2 | 3 | 4 | 5 | 6 | | 7 |
| 42 | | You worried about whether your family members should have genetic tests for cancer. | 1 | 2 | 3 | 4 | 5 | 6 | | 7 |
| 43 | | You had money problems that arose because you had cancer. | 1 | 2 | 3 | 4 | 5 | 6 | | 7 |
| 44 | | You felt people treated you differently because of changes to your appearance due to your cancer or its treatment. | 1 | 2 | 3 | 4 | 5 | 6 | | 7 |
| 45 | | You had financial problems due to a loss of income as a result of cancer. | 1 | 2 | 3 | 4 | 5 | 6 | | 7 |
| 46 | | Whenever you felt a pain, you worried that it might be cancer again. | 1 | 2 | 3 | 4 | 5 | 6 | | 7 |
| 47 | | You were preoccupied with concerns about cancer. | 1 | 2 | 3 | 4 | 5 | 6 | | 7 |

### Appendix 4. Generic domains of HRQoL [[36](#_ENREF_36)]

We would like to ask you about some things that can affect the quality of people’s lives. Some of these questions may sound similar, but please be sure to answer each one. Below is a scale ranging from never to always. Please indicate how often each of these statements has been true for you in the past four weeks. [Select one answer for each question]

|  | | **Never** | **Seldom** | **Sometimes** | **About as often as not** | **Frequently** | **Very often** | **Always** | |
| --- | --- | --- | --- | --- | --- | --- | --- | --- | --- |
| **In the past 4 weeks…** | | | | | | | | | |
| 1 | You had the energy to do the things you wanted to do. | 1 | 2 | 3 | 4 | 5 | 6 | | 7 |
| 2 | You had difficulty doing activities that require concentrating. | 1 | 2 | 3 | 4 | 5 | 6 | | 7 |
| 3 | You were bothered by having a short attention span. | 1 | 2 | 3 | 4 | 5 | 6 | | 7 |
| 4 | You had trouble remembering things. | 1 | 2 | 3 | 4 | 5 | 6 | | 7 |
| 5 | You felt fatigued. | 1 | 2 | 3 | 4 | 5 | 6 | | 7 |
| 6 | You felt happy. | 1 | 2 | 3 | 4 | 5 | 6 | | 7 |
| 7 | You felt blue or depressed. | 1 | 2 | 3 | 4 | 5 | 6 | | 7 |
| 8 | You enjoyed life. | 1 | 2 | 3 | 4 | 5 | 6 | | 7 |
| 9 | You worried about little things. | 1 | 2 | 3 | 4 | 5 | 6 | | 7 |
| 10 | You were bothered by being unable to function sexually. | 1 | 2 | 3 | 4 | 5 | 6 | | 7 |
| 11 | You didn’t have energy to do the things you wanted to do. | 1 | 2 | 3 | 4 | 5 | 6 | | 7 |
| 12 | You were dissatisfied with your sex life. | 1 | 2 | 3 | 4 | 5 | 6 | | 7 |
| 13 | You were bothered by pain that kept you from doing the things you wanted to do. | 1 | 2 | 3 | 4 | 5 | 6 | | 7 |
| 14 | You felt tired a lot. | 1 | 2 | 3 | 4 | 5 | 6 | | 7 |
| 15 | You were reluctant to start new relationships. | 1 | 2 | 3 | 4 | 5 | 6 | | 7 |
| 16 | You lacked interest in sex. | 1 | 2 | 3 | 4 | 5 | 6 | | 7 |
| 17 | Your mood was disrupted by pain or its treatment. | 1 | 2 | 3 | 4 | 5 | 6 | | 7 |
| 18 | You avoided social gatherings. | 1 | 2 | 3 | 4 | 5 | 6 | | 7 |
| 19 | You were bothered by mood swings. | 1 | 2 | 3 | 4 | 5 | 6 | | 7 |
| 20 | You avoided your friends. | 1 | 2 | 3 | 4 | 5 | 6 | | 7 |
| 21 | You had aches or pains. | 1 | 2 | 3 | 4 | 5 | 6 | | 7 |
| 22 | You had a positive outlook on life. | 1 | 2 | 3 | 4 | 5 | 6 | | 7 |
| 23 | You were bothered by forgetting what you started to do. | 1 | 2 | 3 | 4 | 5 | 6 | | 7 |
| 24 | You felt anxious. | 1 | 2 | 3 | 4 | 5 | 6 | | 7 |
| 25 | You were reluctant to meet new people. | 1 | 2 | 3 | 4 | 5 | 6 | | 7 |
| 26 | You avoided sexual activity. | 1 | 2 | 3 | 4 | 5 | 6 | | 7 |
| 27 | Pain or its treatment interfered with your social activities. | 1 | 2 | 3 | 4 | 5 | 6 | | 7 |
| 28 | You were content with your life. | 1 | 2 | 3 | 4 | 5 | 6 | | 7 |

### Appendix 5. Hospital Anxiety and Depression Scale [[37](#_ENREF_37)]

| Hospital Anxiety and Depression Scale (HADS) | | | | 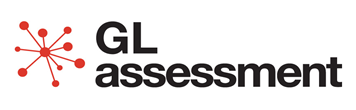 | |
| --- | --- | --- | --- | --- | --- |
| This questionnaire is designed to help clinicians to know how you feel. Read each item below and underline the reply which comes closest to how you have been feeling in the past week.  Don’t take too long over your replies, your immediate reaction to each item will probably be more accurate than a long, thought-out response. | | | | | |
|  | **I feel tense or ‘wound up’** |  | **I feel as if I am slowed down** | |  |
|  | Most of the time |  | Nearly all the time | |  |
|  | A lot of the time |  | Very often | |  |
|  | From time to time, occasionally |  | Sometimes | |  |
|  | Not at all |  | Not at all | |  |
|  | **I still enjoy the things I used to enjoy** |  | **I get a sort of frightened feeling like ‘butterflies’ in the stomach** | |  |
|  | Definitely as much |  | Not at all | |  |
|  | Not quite so much |  | Occasionally | |  |
|  | Only a little |  | Quite often | |  |
|  | Hardly at all |  | Very often | |  |
|  | **I get a sort of frightened feeling as if something awful is about to happen** |  | **I have lost interest in my appearance** | |  |
|  | Very definitely and quite badly |  | Definitely | |  |
|  | Yes, but not too badly |  | I don’t take as much care as I should | |  |
|  | A little, but it doesn’t worry me |  | I may not take quite as much care | |  |
|  | Not at all |  | I take just as much care as ever | |  |
|  | **I can laugh and see the funny side of things** |  | **I feel restless as if I have to be on the move** | |  |
|  | As much as I always could |  | Very much indeed | |  |
|  | Not quite so much now |  | Quite a lot | |  |
|  | Definitely not so much now |  | Not very much | |  |
|  | Not at all |  | Not at all | |  |
|  | **Worrying thoughts go through my mind** |  | **I look forward with enjoyment to things** | |  |
|  | A great deal of the time |  | As much as I ever did | |  |
|  | A lot of the time |  | Rather less than I used to | |  |
|  | Not too often |  | Definitely less than I used to | |  |
|  | Very little |  | Hardly at all | |  |
|  | **I feel cheerful** |  | **I get sudden feelings of panic** | |  |
|  | Never |  | Very often indeed | |  |
|  | Not often |  | Quite often | |  |
|  | Sometimes |  | Not very often | |  |
|  | Most of the time |  | Not at all | |  |
|  | **I can sit at ease and feel relaxed** |  | **I can enjoy a good book or radio or television programme** | |  |
|  | Definitely |  | Often | |  |
|  | Usually |  | Sometimes | |  |
|  | Not often |  | Not often | |  |
|  | Not at all |  | Very seldom | |  |
|  | | | | | |
| **Now check that you have answered all the questions** | | | | | |
|  | | | | | |
| HADS copyright © R.P. Snaith and A.S. Zigmond, 1983, 1992, 1994.  Record form items originally published in *Acta Psychiatrica Scandinavica*, 67, 361–70, copyright © Munksgaard International Publishers Ltd, Copenhagen, 1983. This edition first published in 1994 by nferNelson Publishing Company Ltd, now GL Assessment Limited, 1^st^ Floor Vantage London, Great West Road, Brentford TW8 9AG United Kingdom; GL Assessment is part of GL Education [www.gl-assessment.co.uk](http://www.gl-assessment.co.uk). This form may not be reproduced by any means without first obtaining permission from the publisher. Email: [permissions@gl-assessment.co.uk](mailto:permissions@gl-assessment.co.uk). All rights reserved including translations. | | | | | |
| HADS copyright © R.P. Snaith and A.S. Zigmond, 1983, 1992, 1994.  Record form items originally published in *Acta Psychiatrica Scandinavica*, 67, 361–70,  copyright © Munksgaard International Publishers Ltd, Copenhagen, 1983.  This edition first published in 1994 by nferNelson Publishing Company Ltd, now GL Assessment Limited,  1^st^ Floor Vantage London, Great West Road, Brentford TW8 9AG United Kingdom  GL Assessment is part of GL Education [www.gl-assessment.co.uk](http://www.gl-assessment.co.uk)  This form may not be reproduced by any means without first obtaining permission from the publisher.  Email: [permissions@gl-assessment.co.uk](mailto:permissions@gl-assessment.co.uk)  All rights reserved including translations | | | | | |

### Appendix 6. Clinical information

| **1. What treatments have you received for your breast cancer? (Tick all that apply)** | |
| --- | --- |
| □ | Surgery |
| □ | Radiotherapy |
| □ | Chemotherapy (excluding hormone treatment) |
| □ | Hormone treatment |
| □ | Monoclonal antibodies / immunotherapy |
| □ | Don’t know / can’t remember |
|  |  |
| **1.1 If you have had breast surgery, do any of the following apply to you? (Tick all that apply)** | |
| □ | I have had a lumpectomy (partial removal of the breast) |
| □ | I have had a mastectomy (complete removal of the breast) |
| □ | I have had a bilateral mastectomy (complete removal of the two breasts) |
| □ | I have had a breast reconstruction |
| □ | I am awaiting or considering breast reconstruction |
| □ | None of these apply to me |
| □ | Don’t know / can’t remember |
|  |  |
| **2. At the time of the diagnosis, your cancer was:** | |
| □ | Localised to the breast only (without involving lymph nodes) |
| □ | Spread to the lymph nodes in the axilla |
| □ | Spread beyond the breast and the lymph nodes (metastatic) |
| □ | Don’t know / can’t remember |
|  |  |
| **2.1 Please select your stage at diagnosis:** | |
| □ | Stage I |
| □ | Stage II |
| □ | Stage III |
| □ | Stage IV |
| □ | Don’t know / can’t remember |
|  |  |
| **3. How long is it since you completed your initial cancer treatment?**  (Treatment includes any chemotherapy, radiotherapy or surgery for your breast cancer. When answering this question please do not include hormone treatments such as Tamoxifen.) | |
| □ | I am still having my initial treatment |
| □ | It is less than 3 months since my initial treatment |
| □ | It is between 3 and 12 months since my initial treatment |
| □ | It is between 1 and 5 years since my initial treatment |
| □ | It is more than 5 years since my initial treatment |
| □ | Don’t know / can’t remember |
|  |  |
| **4. Regarding your menopausal status before and after the breast cancer diagnosis:** | |
| □ | My menstrual periods had finished when my cancer was diagnosed |
| □ | My menstrual periods finished during my treatments for breast cancer |
| □ | I had periods before the cancer diagnosis and continued to have them during/after the treatments |
| □ | Don’t know / can’t remember |
|  |  |
| **5. Is your cancer currently in remission?** (Complete remission means that there is no sign of cancer in your body) | |
| □ | Yes |
| □ | No |
| □ | Don’t know |

### Appendix 7. Demographic information

| **1. Which of these qualifications do you have?** | |
| --- | --- |
| □ | Up to GCSEs, O levels, or equivalent |
| □ | A levels or equivalent |
| □ | Undergraduate degree (for example BA, BSc) |
| □ | Post-graduate degree |
| □ | Trade, technical or vocational training |
| □ | Do not wish to disclose |
|  |  |
| **2. What is your ethnic group?** | |
| □ | White |
| □ | Mixed / Multiple ethnic groups |
| □ | Asian / Asian British |
| □ | Black / African / Caribbean / Black British |
| □ | Other ethnic group |
| □ | Do not wish to disclose |
|  |  |
| **3. Which statement best describes your living arrangements?** | |
| □ | I live with partner / spouse |
| □ | I live with family / friends |
| □ | I live alone |
| □ | I live in a nursing home or other long term care home |
| □ | Other |
| □ | Do not wish to disclose |
|  |  |

# **Supplementary table 1** Comparison between participants and non-participants by exposure group, age group, country, and deprivation.

|  |  | **Women with no history of cancer**  **(Number invited = 1,460)** | | | |  | **Breast cancer survivors**  **(Number invited = 1,018)** | | | |
| --- | --- | --- | --- | --- | --- | --- | --- | --- | --- | --- |
|  |  | **Refused** | | **Participated** | |  | **Refused** | | **Participated** | |
|  |  | **No.** | **%** | **No.** | **%** |  | **No.** | **%** | **No.** | **%** |
|  |  |  |  |  |  |  |  |  |  |  |
| **Total** | | 1,208 | 82.7 | 252 | 17.3 |  | 665 | 64.3 | 353 | 34.7 |
|  |  |  |  |  |  |  |  |  |  |  |
| **Age (years)** | |  |  |  |  |  |  |  |  |  |
|  | 18-39 | 17 | 1.4 | 1 | 0.4 |  | 5 | 0.8 | 2 | 0.6 |
|  | 40-49 | 107 | 8.9 | 16 | 6.4 |  | 45 | 6.8 | 19 | 5.4 |
|  | 50-59 | 263 | 21.8 | 54 | 21.4 |  | 168 | 25.3 | 79 | 22.4 |
|  | 60-69 | 355 | 29.4 | 80 | 31.8 |  | 200 | 30.1 | 130 | 36.8 |
|  | 70-81 | 466 | 38.6 | 101 | 40.1 |  | 247 | 37.1 | 123 | 34.8 |
|  |  |  |  |  |  |  |  |  |  |  |
| **Country** | |  |  |  |  |  |  |  |  |  |
|  | England | 233 | 19.3 | 64 | 25.4 |  | 95 | 14.3 | 50 | 14.2 |
|  | Northern Ireland | 239 | 19.8 | 16 | 6.4 |  | 83 | 12.5 | 33 | 9.4 |
|  | Scotland | 356 | 29.5 | 74 | 29.4 |  | 241 | 36.2 | 114 | 32.3 |
|  | Wales | 380 | 31.5 | 98 | 38.9 |  | 246 | 37.0 | 156 | 44.2 |
|  |  |  |  |  |  |  |  |  |  |  |
| **IMD quintile** | |  |  |  |  |  |  |  |  |  |
|  | 1 (most deprived) | 209 | 17.3 | 53 | 21.0 |  | 97 | 14.6 | 71 | 20.1 |
|  | 2 | 239 | 19.8 | 36 | 14.3 |  | 97 | 14.6 | 54 | 15.3 |
|  | 3 | 138 | 11.4 | 26 | 10.3 |  | 123 | 18.5 | 53 | 15.0 |
|  | 4 | 440 | 36.4 | 98 | 38.9 |  | 277 | 41.7 | 140 | 39.7 |
|  | 5 (least deprived) | 182 | 15.1 | 39 | 15.5 |  | 71 | 10.7 | 35 | 9.9 |
|  |  |  |  |  |  |  |  |  |  |  |

IMD = Index of Multiple Deprivation; No = number.

# **Supplementary table 2** Associations between age and quality of life, anxiety and depression in breast cancer survivors (N=353).

|  | | | **Age** | | | | | | | | | | | | | | | | | |
| --- | --- | --- | --- | --- | --- | --- | --- | --- | --- | --- | --- | --- | --- | --- | --- | --- | --- | --- | --- | --- |
|  |  |  | **Unadjusted association** | | | | |  | **Model 1:**  **adjusted for socio-demographic variables^1^** | | | | |  | **Model 2:**  **model 1 + stage & chemotherapy** | | | | |  |
|  |  |  | **<60 yrs** | **60-69 yrs** | | **70-81 yrs** | |  | **<60 yrs** | **60-69 yrs** | | **70-81 yrs** | |  | **<60 yrs** | **60-69 yrs** | | **70-81 yrs** | |  |
|  |  |  |  | **β** | **95%CI** | **β** | **95%CI** |  |  | **β** | **95%CI** | **β** | **95%CI** |  |  | **β** | **95%CI** | **β** | **95%CI** | |
| **Quality of Life in Adult Cancer Survivors Scale** | | | | | | | |  |  |  |  |  |  |  |  |  |  |  |  | |
|  | **Generic domains** | |  |  |  |  |  |  |  |  |  |  |  |  |  |  |  |  |  | |
|  |  | Negative feelings | Ref | **-1.0** | **(-2.4: -0.4)** | **-2.0** | **(-3.9: -0.1)** |  | Ref | -1.4 | (-2.8: 0.1) | **-2.4** | **(-4.2: -0.6)** |  | Ref | -1.3 | (-2.8: 0.2) | **-2.2** | **(-4.1: -0.3)** | |
|  |  | Positive feelings | Ref | 0.1 | (-0.8: 1.1) | 1.1 | (-0.5: 2.8) |  | Ref | 0.4 | (-0.8: 1.7) | 1.7 | (-0.1: 3.4) |  | Ref | 0.3 | (-1.0: 1.6) | 1.5 | (-0.3: 3.3) | |
|  |  | Cognitive problems | Ref | -1.0 | (-2.4: 0.3) | **-2.7** | **(-4.1: -1.2)** |  | Ref | -1.6 | (-3.1: -0.1) | **-2.7** | **(-4.3: -1.1)** |  | Ref | -1.3 | (-2.9: 0.3) | **-2.2** | **(-3.8: -0.6)** | |
|  |  | Pain | Ref | 0.1 | (-1.6: 1.8) | -0.6 | (-2.7: 1.5) |  | Ref | -0.1 | (-1.9: 1.6) | -0.8 | (-2.8: 1.2) |  | Ref | 0.03 | (-1.7: 1.7) | -0.7 | (-3.0: 1.5) | |
|  |  | Sexual function | Ref | -2.0 | (-4.4: 0.3) | **-3.3** | **(-5.2: -1.3)** |  | Ref | -3.3 | (-5.7: -1.0) | **-4.3** | **(-6.2: -2.5)** |  | Ref | -3.2 | (-5.6: -0.9) | **-4.4** | **(-6.4: -2.4)** | |
|  |  | Energy/Fatigue | Ref | -0.3 | (-1.6: 0.9) | -0.3 | (-1.9: 1.2) |  | Ref | -0.8 | (-2.3: 0.7) | -0.7 | (-2.3: 0.9) |  | Ref | -0.7 | (-2.2: 0.7) | -0.8 | (-2.5: 0.9) | |
|  |  | Avoidance | Ref | -0.6 | (-2.2: 1.0) | **-2.3** | **(-4.2: -0.4)** |  | Ref | -1.1 | (-2.8: 0.6) | **-2.4** | **(-4.4: -0.5)** |  | Ref | -1.0 | (-2.7: 0.7) | **-2.3** | **(-4.4: -0.2)** | |
|  |  | Summary score | Ref | -4.5 | (-13.0: 3.9) | **-12.6** | **(-22.5: -2.7)** |  | Ref | -8.9 | (-17.9: -0.02) | **-15.5** | **(-25.1: -5.9)** |  | Ref | -8.0 | (-16.9: 0.9) | **-14.7** | **(-24.8: -4.5)** | |
|  | **Cancer specific domains** | |  |  |  |  |  |  |  |  |  |  |  |  |  |  |  |  |  | |
|  |  | Financial problems | Ref | **-2.5** | **(-4.3: -0.8)** | **-3.2** | **(-4.8: -1.5)** |  | Ref | **-2.5** | **(-4.4: -0.5)** | **-3.1** | **(-4.7: -1.5)** |  | Ref | **-2.3** | **(-4.3: -0.4)** | **-2.8** | **(-4.4: -1.1)** | |
|  |  | Benefits | Ref | -1.6 | (-3.5: 0.3) | 0.6 | (-1.6: 2.7) |  | Ref | -1.0 | (-2.8: 0.9) | 0.4 | (-1.9: 2.7) |  | Ref | -0.9 | (-2.8: 0.9) | 0.3 | (-2.0: 2.7) | |
|  |  | Distress-family | Ref | 0.2 | (-1.8: 2.2) | -0.3 | (-2.2: 1.5) |  | Ref | 0.5 | (-1.3: 2.3) | -1.2 | (-2.7: 0.3) |  | Ref | 0.5 | (-1.4: 2.4) | -1.1 | (-2.9: 0.6) | |
|  |  | Appearance | Ref | -1.3 | (-2.9: 0.3) | **-3.4** | **(-4.9: -1.9)** |  | Ref | **-1.7** | **(-3.4: -0.1)** | **-4.2** | **(-5.9: -2.5)** |  | Ref | -1.2 | (-2.9: 0.6) | **-3.1** | **(-4.7: -1.5)** | |
|  |  | Distress-recurrence | Ref | -1.5 | (-3.5: 0.6) | **-3.2** | **(-5.1: -1.4)** |  | Ref | **-1.7** | **(-3.8: 0.4)** | **-3.6** | **(-5.7: -1.5)** |  | Ref | -1.3 | (-3.4: 0.8) | **-2.8** | **(-4.8: -0.7)** | |
|  |  | Summary score | Ref | -4.9 | (-10.7: 0.9) | **-10.0** | **(-15.3: -4.7)** |  | Ref | **-5.4** | **(-11.3: 0.4)** | **-12.1** | **(-17.3: -6.9)** |  | Ref | -4.3 | (-10.5: 1.8) | **-9.8** | **(-15.3: -4.4)** | |
|  |  |  |  |  |  |  |  |  |  |  |  |  |  |  |  |  |  |  |  | |
| **Hospital Anxiety and Depression Scale** | | | | | | | | |  |  |  |  |  |  |  |  |  |  |  | |
|  |  | Anxiety | Ref | -1.2 | (-2.5: 0.1) | **-1.9** | **(-3.2: -0.5)** |  | Ref | -1.1 | (-2.4: 0.3) | **-1.9** | **(-3.3: -0.5)** |  | Ref | -1.0 | (-2.4: 0.5) | **-1.7** | **(-3.1: -0.2)** | |
|  |  | Depression | Ref | -0.5 | (-1.4: 0.4) | -0.8 | (-2.1: 0.4) |  | Ref | -0.6 | (-1.7: 0.4) | **-1.3** | **(-2.4: -0.1)** |  | Ref | -0.6 | (-1.7: 0.5) | **-1.3** | **(-2.5: -0.1)** | |

^1^ Adjusted for education (university degree vs. no degree), practice postcode quintile of the index of multiple deprivation (IMD), and country (Scotland, Wales, Northern Ireland, England).

Ref = reference category; Yrs = years; 95%CI = 95% Confidence interval.

# **Supplementary table 3** Associations between menopausal status at cancer diagnosis and quality of life, anxiety and depression in breast cancer survivors (N=353).

|  | | | **Menopausal status** | | | | | | | | | | | | | |
| --- | --- | --- | --- | --- | --- | --- | --- | --- | --- | --- | --- | --- | --- | --- | --- | --- |
|  |  |  | **Unadjusted association** | | |  | **Model 1: adjusted for**  **socio-demographic variables^1^** | | | |  | **Model 2:**  **model 1 + stage & chemotherapy** | | | | |
|  |  |  | **Menopausal** | **Not menopausal** | |  | **Menopausal** | **Not menopausal** | | |  | **Menopausal** | **Not menopausal** | | | |
|  |  |  |  | **β** | **95%CI** |  |  | **β** | **95%CI** | |  |  | **β** | | **95%CI** | |
| **Quality of Life in Adult Cancer Survivors Scale** | | | | | |  |  |  |  |  | |  | |  | |  |
|  | **Generic domains** | |  |  |  |  |  |  |  | |  |  |  | |  | |
|  |  | Negative feelings | Ref | **1.8** | **(0.6: 3.0)** |  | Ref | **2.0** | **(0.7: 3.3)** | |  | Ref | **1.8** | | **(0.3: 3.3)** | |
|  |  | Positive feelings | Ref | -1.1 | (-2.3: 0.1) |  | Ref | -1.3 | (-2.9: 0.2) | |  | Ref | -1.4 | | (-2.9: 0.1) | |
|  |  | Cognitive problems | Ref | **1.4** | **(0.1: 2.8)** |  | Ref | 1.4 | (-0.04: 2.8) | |  | Ref | 1.0 | | (-0.5: 2.5) | |
|  |  | Pain | Ref | -0.6 | (-2.0: 0.9) |  | Ref | -0.3 | (-2.0: 1.5) | |  | Ref | -0.7 | | (-2.4: 1.1) | |
|  |  | Sexual function | Ref | **1.9** | **(0.2: 3.7)** |  | Ref | 0.9 | (-0.9: 2.8) | |  | Ref | 0.7 | | (-1.3: 2.6) | |
|  |  | Energy/Fatigue | Ref | 0.3 | (-0.9: 1.5) |  | Ref | 0.6 | (-0.6: 1.7) | |  | Ref | 0.4 | | (-0.8: 1.6) | |
|  |  | Avoidance | Ref | **1.7** | **(0.2: 3.1)** |  | Ref | **2.0** | **(0.3: 3.6)** | |  | Ref | **1.8** | | **(0.2: 3.5)** | |
|  |  | Summary score | Ref | **8.1** | **(1.8: 14.4)** |  | Ref | 7.9 | (-0.1: 15.9) | |  | Ref | 6.0 | | (-2.5:14.4) | |
|  | **Cancer-specific domains** | |  |  |  |  |  |  |  | |  |  |  | |  | |
|  |  | Financial problems | Ref | **1.7** | **(0.3: 3.1)** |  | Ref | 0.5 | (-1.2: 2.2) | |  | Ref | 0.2 | | (-1.5: 1.8) | |
|  |  | Benefits | Ref | 0.4 | (-1.2: 2.0) |  | Ref | 0.5 | (-1.2: 2.2) | |  | Ref | 0.2 | | (-1.4: 1.8) | |
|  |  | Distress-family | Ref | 0.6 | (-1.4: 2.6) |  | Ref | 0.9 | (-1.6: 3.4) | |  | Ref | 0.6 | | (-2.0: 3.3) | |
|  |  | Appearance | Ref | **2.3** | **(1.1: 3.4)** |  | Ref | 1.6 | (-0.3: 3.6) | |  | Ref | 0.8 | | (-1.2: 2.8) | |
|  |  | Distress-recurrence | Ref | **2.0** | **(0.5: 3.4)** |  | Ref | 1.1 | (-1.3: 3.6) | |  | Ref | 0.5 | | (-2.0: 3.1) | |
|  |  | Summary score | Ref | **6.6** | **(2.2: 11.1)** |  | Ref | 4.2 | (-2.5: 10.9) | |  | Ref | 2.1 | | (-4.7: 9.0) | |
| **Hospital Anxiety and Depression Scale** | | | |  |  |  |  |  |  | |  |  |  | |  | |
|  |  | Anxiety | Ref | **1.9** | **(0.8: 3.0)** |  | Ref | **1.7** | **(0.6: 2.8)** | |  | Ref | **1.6** | | **(0.5: 2.7)** | |
|  |  | Depression | Ref | 0.5 | (-0.5: 1.6) |  | Ref | 0.5 | (-0.6: 1.5) | |  | Ref | 0.5 | | (-0.5: 1.5) | |

^1^ Adjusted for age (<60, 60-69, 70-81 years), education (higher education degree vs. no degree), practice postcode quintile level of the index of multiple deprivation (IMD), and country (Scotland, Wales, Northern Ireland, England).

Ref = reference category; 95%CI = 95% Confidence interval.

# **Supplementary table 4** Associations between exposure to chemotherapy and quality of life, anxiety and depression in breast cancer survivors (N=353).

|  | | | **Chemotherapy exposure** | | | | | | | | | | |
| --- | --- | --- | --- | --- | --- | --- | --- | --- | --- | --- | --- | --- | --- |
|  |  |  | **Unadjusted association** | | |  | **Model 1:**  **adjusted for socio-demographic variables^1^** | | |  | **Model 2:**  **model 1 + stage at diagnosis** | | |
|  |  |  | **No ChemoT** | **ChemoT** | |  | **No ChemoT** | **ChemoT** | |  | **No ChemoT** | **ChemoT** | |
|  |  |  |  | **β** | **95%CI** |  |  | **β** | **95%CI** |  |  | **β** | **95%CI** |
| **Quality of Life in Adult Cancer Survivors Scale** | | | | | |  |  |  |  |  |  |  |  |
|  | **Generic domains** | |  |  |  |  |  |  |  |  |  |  |  |
|  |  | Negative feelings | Ref | **1.7** | **(0.6: 2.8)** |  | Ref | 1.3 | (-0.01: 2.7) |  | Ref | 1.0 | (-0.4: 2.5) |
|  |  | Positive feelings | Ref | -0.7 | (-1.8: 0.5) |  | Ref | -0.4 | (-1.7: 0.9) |  | Ref | -0.4 | (-1.8: 0.9) |
|  |  | Cognitive problems | Ref | **2.0** | **(0.9: 3.1)** |  | Ref | **1.9** | **(0.7: 3.1)** |  | Ref | **1.6** | **(0.2: 3.0)** |
|  |  | Pain | Ref | 0.7 | (-0.5: 2.0) |  | Ref | 0.8 | (-0.9: 2.5) |  | Ref | -0.2 | (-2.1: 1.8) |
|  |  | Sexual function | Ref | **1.6** | **(0.3: 2.9)** |  | Ref | 1.0 | (-0.6: 2.6) |  | Ref | 0.01 | (-1.9: 1.9) |
|  |  | Energy/Fatigue | Ref | 0.1 | (-0.7: 1.0) |  | Ref | 0.3 | (-0.6: 1.3) |  | Ref | -0.2 | (-1.3: 0.9) |
|  |  | Avoidance | Ref | **1.4** | **(0.4: 2.5)** |  | Ref | 1.0 | (-0.2: 2.2) |  | Ref | 0.7 | (-0.6: 2.0) |
|  |  | Summary score | Ref | **8.7** | **(3.8: 13.7)** |  | Ref | **7.8** | **(1.2: 14.3)** |  | Ref | 4.0 | (-3.5: 11.6) |
|  | **Cancer specific domains** | |  |  |  |  |  |  |  |  |  |  |  |
|  |  | Financial problems | Ref | **2.0** | **(0.7: 3.4)** |  | Ref | 1.4 | (-0.1: 2.8) |  | Ref | 1.2 | (-0.6: 3.0) |
|  |  | Benefits | Ref | 0.9 | (-0.6: 2.4) |  | Ref | 0.4 | (-1.3: 2.2) |  | Ref | 0.03 | (-2.0: 2.1) |
|  |  | Distress-family | Ref | 1.0 | (-0.2: 2.2) |  | Ref | 1.2 | (-0.3: 2.6) |  | Ref | 0.8 | (-0.7: 2.3) |
|  |  | Appearance | Ref | **4.4** | **(3.1: 5.7)** |  | Ref | **4.1** | **(2.7: 5.6)** |  | Ref | **3.5** | **(1.8: 5.2)** |
|  |  | Distress-recurrence | Ref | **3.2** | **(2.1: 4.3)** |  | Ref | **3.1** | **(1.9: 4.3)** |  | Ref | **2.9** | **(1.4: 4.5)** |
|  |  | Summary score | Ref | **10.9** | **(7.1: 14.7)** |  | Ref | **9.8** | **(5.5: 14.1)** |  | Ref | **8.4** | **(3.4: 13.4)** |
| **Hospital Anxiety and Depression Scale** | | | |  |  |  |  |  |  |  |  |  |  |
|  |  | Anxiety | Ref | **1.1** | **(0.2: 2.0)** |  | Ref | 0.9 | (-0.2: 2.0) |  | Ref | 0.9 | (-2.4: 0.5) |
|  |  | Depression | Ref | 0.2 | (-0.6: 0.9) |  | Ref | -0.1 | (-1.0: 0.8) |  | Ref | -0.3 | (-1.2: 0.7) |

^1^ Adjusted for age (34-59, 60-69, 70-81 years), education (graduate degree: yes/no), practice postcode quintile of the index of multiple deprivation (IMD), and country (Scotland, Wales, Northern Ireland, England).

Ref = reference category; 95%CI = 95% Confidence interval.

# **Supplementary table 5** Associations between stage of the disease at diagnosis and quality of life, anxiety and depression in breast cancer survivors (N=353).

|  | | | **Stage at diagnosis** | | | | | | | | | | | | | | | |  |
| --- | --- | --- | --- | --- | --- | --- | --- | --- | --- | --- | --- | --- | --- | --- | --- | --- | --- | --- | --- |
|  |  |  | **Unadjusted association** | | |  | **Model 1:**  **adjusted for socio-demographic variables^1^** | | | |  | | **Model 2:**  **model 1 + chemotherapy** | | | | | |  |
|  |  |  | **Early**  **stage** | **Regional or**  **distant metastases** | |  | **Early**  **stage** | **Regional or distant metastases** | | |  | | **Early**  **stage** | | **Regional or**  **distant metastases** | | | |  |
|  |  |  |  | **β** | **95%CI** |  |  | **β** | **95%CI** | |  | |  | | **β** | | **95%CI** | |  |
| **Quality of Life in Adult Cancer Survivors Scale** | | | | | |  |  |  | |  | |  | |  | |  | |  | |
|  | **Generic domains** | |  |  |  |  |  |  |  | |  | |  | |  | |  |  |  |
|  |  | Negative feelings | Ref | 1.2 | (-0.1: 2.6) |  | Ref | **1.2** | **(0.1: 2.4)** | |  | | Ref | | 0.7 | | (-0.5: 2.0) | |  |
|  |  | Positive feelings | Ref | -0.3 | (-1.8: 1.1) |  | Ref | -0.2 | (-1.5: 1.1) | |  | | Ref | | -0.01 | | (-1.4: 1.4) | |  |
|  |  | Cognitive problems | Ref | **1.2** | **(0.3: 2.1)** |  | Ref | **1.2** | **(0.2: 2.2)** | |  | | Ref | | 0.5 | | (-0.7: 1.6) | |  |
|  |  | Pain | Ref | **1.9** | **(0.7: 3.2)** |  | Ref | **1.9** | **(0.4: 3.3)** | |  | | Ref | | **1.9** | | **(0.3: 3.6)** | |  |
|  |  | Sexual function | Ref | **2.0** | **(0.5: 3.5)** |  | Ref | **2.1** | **(0.6: 3.5)** | |  | | Ref | | **2.1** | | **(0.4: 3.7)** | |  |
|  |  | Energy/Fatigue | Ref | 0.8 | (-0.3: 1.9) |  | Ref | 0.9 | (-0.3: 2.0) | |  | | Ref | | 1.0 | | (-0.3: 2.2) | |  |
|  |  | Avoidance | Ref | 1.1 | (-0.2: 2.4) |  | Ref | 1.0 | (-0.3: 2.2) | |  | | Ref | | 0.6 | | (-0.7: 2.0) | |  |
|  |  | Summary score | Ref | **8.6** | **(2.4: 14.8)** |  | Ref | **9.7** | **(3.6: 15.9)** | |  | | Ref | | **7.9** | | **(0.9: 14.9)** | |  |
|  | **Cancer specific domains** | |  |  |  |  |  |  |  | |  | |  | |  | |  | |  |
|  |  | Financial problems | Ref | **1.3** | **(0.3: 2.2)** |  | Ref | 0.8 | (-0.1: 1.8) | |  | | Ref | | 0.3 | | (-0.9: 1.5) | |  |
|  |  | Benefits | Ref | 0.3 | (-0.9: 1.5) |  | Ref | 0.3 | (-0.9: 1.6) | |  | | Ref | | 0.3 | | (-1.1: 1.8) | |  |
|  |  | Distress-family | Ref | 0.8 | (-1.1: 2.7) |  | Ref | 1.1 | (-0.9: 3.1) | |  | | Ref | | 0.8 | | (-1.4: 3.0) | |  |
|  |  | Appearance | Ref | **2.6** | **(1.4: 3.8)** |  | Ref | **2.7** | **(1.4: 4.1)** | |  | | Ref | | 1.2 | | (-0.4: 2.7) | |  |
|  |  | Distress-recurrence | Ref | **1.7** | **(0.02: 3.3)** |  | Ref | 1.5 | (-0.1: 3.1) | |  | | Ref | | 0.2 | | (-1.9: 2.2) | |  |
|  |  | Summary score | Ref | **6.3** | **(1.6: 11.0)** |  | Ref | **6.2** | **(1.8: 10.7)** | |  | | Ref | | 2.4 | | (-2.8: 7.6) | |  |
| **Hospital Anxiety and Depression Scale** | | | |  |  |  |  |  |  | |  | |  | |  | |  | |  |
|  |  | Anxiety | Ref | 0.5 | (-0.7: 1.7) |  | Ref | 0.5 | (-0.7: 1.6) | |  | | Ref | | 0.1 | | (-1.1: 1.3) | |  |
|  |  | Depression | Ref | 0.5 | (-0.4: 1.3) |  | Ref | 0.4 | (-0.4: 1.2) | |  | | Ref | | 0.5 | | (-0.4: 1.4) | |  |

^1^ Adjusted for age (34-59, 60-69, 70-81 years), practice postcode quintile level of the index of multiple deprivation (IMD), and country (Scotland, Wales, Northern Ireland, England).

Ref = reference category; 95%CI = 95% Confidence interval.

# **Supplementary table 6** Associations between living arrangements and quality of life, anxiety and depression in breast cancer survivors (N=353).

|  | | | **Living arrangements** | | | | | | | | | | | |
| --- | --- | --- | --- | --- | --- | --- | --- | --- | --- | --- | --- | --- | --- | --- |
|  |  |  | **Unadjusted association** | | |  | **Model 1: adjusted for**  **socio-demographic variables^1^** | | | |  | **Model 2:**  **model 1 + stage and chemotherapy** | | |
|  |  |  | **Living alone** | **Not alone** | |  | **Living alone** | **Not alone** | | |  | **Living alone** | **Not alone** | |
|  |  |  |  | **β** | **95%CI** |  |  | **β** | | **95%CI** |  |  | **β** | **95%CI** |
| **Quality of Life in Adult Cancer Survivors Scale** | | | | | |  |  |  |  | |  |  |  |  |
|  | **Generic domains** | |  |  |  |  |  |  | |  |  |  |  |  |
|  |  | Negative feelings | Ref | -0.8 | (-2.1; 0.6) |  | Ref | -0.8 | | (-2.1; 0.4) |  | Ref | -0.8 | (-2.1; 0.6) |
|  |  | Positive feelings | Ref | 0.6 | (-0.8: 2.1) |  | Ref | 0.8 | | (-0.8: 2.4) |  | Ref | 0.9 | (-0.8: 2.6) |
|  |  | Cognitive problems | Ref | -1.2 | (-2.6: 0.2) |  | Ref | -0.9 | | (-2.4: 0.6) |  | Ref | -0.8 | (-2.4: 0.9) |
|  |  | Pain | Ref | 0.1 | (-1.8: 2.0) |  | Ref | 0.1 | | (-1.9: 2.1) |  | Ref | 0.1 | (-2.0: 2.2) |
|  |  | Sexual function | Ref | **-2.9** | **(-4.7: -1.2)** |  | Ref | **-2.8** | | **(-4.7: -0.8)** |  | Ref | -2.8 | (4.7: -1.0) |
|  |  | Energy/Fatigue | Ref | -0.3 | (-1.7: 1.1) |  | Ref | -0.3 | | (-1.8: 1.2) |  | Ref | -0.1 | (-1.8: 1.5) |
|  |  | Avoidance | Ref | 0.8 | (-0.7: 2.3) |  | Ref | 0.8 | | (-0.7: 2.4) |  | Ref | 1.0 | (-0.6: 2.6) |
|  |  | Summary score | Ref | -5.9 | (-15.0: 3.2) |  | Ref | -5.7 | | (-14.9: 3.4) |  | Ref | -5.8 | (-14.8: 3.2) |
|  | **Cancer-specific domains** | |  |  |  |  |  |  | |  |  |  |  |  |
|  |  | Financial problems | Ref | -0.2 | (-1.5: 1.0) |  | Ref | 0.2 | | (-1.3: 1.8) |  | Ref | 0.4 | (-1.2: 2.0) |
|  |  | Benefits | Ref | -1.0 | (-3.0: 1.1) |  | Ref | -0.8 | | (-3.0: 1.4) |  | Ref | -0.2 | (-2.6: 2.1) |
|  |  | Distress-family | Ref | -0.6 | (-2.8: 1.5) |  | Ref | -0.4 | | (2.6: 1.8) |  | Ref | -0.2 | (-2.5: 2.1) |
|  |  | Appearance | Ref | **-1.5** | **(-2.7: -0.2)** |  | Ref | -0.8 | | (-2.2: 0.6) |  | Ref | -0.6 | (-1.7: 0.6) |
|  |  | Distress-recurrence | Ref | **-2.7** | **(-4.0: -1.5)** |  | Ref | **-2.4** | | **(-4.1: -0.7)** |  | Ref | **-2.0** | **(-3.9: -0.1)** |
|  |  | Summary score | Ref | **-5.4** | **(-9.8: -1.1)** |  | Ref | -3.4 | | (-8.6: 1.8) |  | Ref | -2.4 | (-7.7: 2.9) |
| **Hospital Anxiety and Depression Scale** | | | |  |  |  |  |  | |  |  |  |  |  |
|  |  | Anxiety | Ref | -0.7 | (-2.0: 0.5) |  | Ref | -0.8 | | (-2.1: 0.6) |  | Ref | -0.7 | (-2.1: 0.7) |
|  |  | Depression | Ref | -0.2 | (-1.3: 0.8) |  | Ref | -0.2 | | (-1.3: 0.8) |  | Ref | -0.3 | (-1.4: 0.8) |

^1^ Adjusted for age (<60, 60-69, 70-81 years), education (higher education degree vs. no degree), practice postcode quintile of the index of multiple deprivation (IMD), and country (Scotland, Wales, Northern Ireland, England).

Ref = reference category; 95%CI = 95% Confidence interval.

# **Supplementary table 7** Associations between education and quality of life, anxiety and depression in breast cancer survivors (N=353).

|  | | | **Education** | | | | | | | | | | | | | | | |  |
| --- | --- | --- | --- | --- | --- | --- | --- | --- | --- | --- | --- | --- | --- | --- | --- | --- | --- | --- | --- |
|  |  |  | **Unadjusted association** | | |  | | **Model 1: adjusted for**  **socio-demographic variables^1^** | | | |  | | | **Model 2:**  **model 1 + stage & chemotherapy** | | | | |
|  |  |  | **No higher education** | **Higher education** | |  | | **No higher education** | **Higher education** | | |  | | | **No higher education** | | **Higher education** | | |
|  |  |  |  | **β** | **95%CI** |  | |  | **β** | **95%CI** | |  | | |  | | **β** | **95%CI** | |
| **Quality of Life in Adult Cancer Survivors Scale** | | | | | |  | |  |  |  |  | | |  | | |  |  | |
|  | **Generic domains** | |  |  |  |  | |  |  |  | |  | | |  | |  |  | |
|  |  | Negative feelings | Ref | -0.7 | (-2.1: 0.7) |  | | Ref | -0.8 | (-2.1: 0.5) | |  | | | Ref | | -0.8 | (-2.1: 0.5) | |
|  |  | Positive feelings | Ref | 0.5 | (-1.1: 2.1) |  | | Ref | 0.5 | (-1.2: 2.2) | |  | | | Ref | | 0.4 | (-1.4: 2.2) | |
|  |  | Cognitive problems | Ref | -0.7 | (-1.9: 0.5) |  | | Ref | -0.9 | (-2.1: 0.4) | |  | | | Ref | | -1.0 | (-2.2: 0.3) | |
|  |  | Pain | Ref | **-1.8** | **(-3.1: -0.4)** |  | | Ref | **-1.8** | **(-3.2: -0.4)** | |  | | | Ref | | **-2.0** | **(-3.4: -0.5)** | |
|  |  | Sexual function | Ref | -1.0 | (-3.0: 0.9) |  | | Ref | -1.3 | (-3.2: 0.6) | |  | | | Ref | | -1.3 | (-3.2: 0.6) | |
|  |  | Energy/Fatigue | Ref | -1.1 | (-2.4: 0.2) |  | | Ref | -1.2 | (-2.6: 0.1) | |  | | | Ref | | -1.3 | (-2.7: 0.1) | |
|  |  | Avoidance | Ref | -0.3 | (-1.7: 1.0) |  | | Ref | -0.4 | (-1.8: 0.9) | |  | | | Ref | | -0.4 | (-1.7: 1.0) | |
|  |  | Summary score | Ref | -6.3 | (-14.5: 2.0) |  | | Ref | -7.1 | (-15.3: 1.03) | |  | | | Ref | | -7.2 | (-15.4: 1.1) | |
|  | **Cancer-specific domains** | |  |  |  |  | |  |  |  | |  | | |  | |  |  | |
|  |  | Financial problems | Ref | -0.6 | (-2.0: 0.7) |  | | Ref | -0.8 | (-2.2: 0.6) | |  | | | Ref | | -0.9 | (-2.3: 0.6) | |
|  |  | Benefits | Ref | -0.8 | (-2.4: 0.8) |  | | Ref | -1.1 | (-2.7: 0.6) | |  | | | Ref | | -1.4 | (-3.1: 0.3) | |
|  |  | Distress-family | Ref | **-3.0** | **(-4.5: -1.4)** |  | | Ref | **-2.9** | **(-4.5: -1.3)** | |  | | | Ref | | **-2.0** | **(-4.6: -1.3)** | |
|  |  | Appearance | Ref | -0.7 | (-2.3: 1.0) |  | | Ref | -1.0 | (-2.6: 0.6) | |  | | | Ref | | -1.1 | (-2.8: 0.5) | |
|  |  | Distress-recurrence | Ref | -1.1 | (-2.9: 0.5) |  | | Ref | -1.4 | (-3.2: 0.3) | |  | | | Ref | | -1.7 | (-3.4: 0.1) | |
|  |  | Summary score | Ref | **-5.4** | **(-10.3: -0.5)** |  | | Ref | **-6.1** | **(-11.1: -1.2)** | |  | | | Ref | | **-6.6** | **(-11.7: -1.4)** | |
| **Hospital Anxiety and Depression Scale** | | | |  |  |  |  | |  |  | | |  | | |  |  |  | |
|  |  | Anxiety | Ref | 0.4 | (-1.0: 1.8) |  | | Ref | 0.3 | (-1.1: 1.7) | |  | | | Ref | | 0.3 | (-1.1: 1.7) | |
|  |  | Depression | Ref | -0.8 | (-1.6: 0.1) |  | | Ref | -0.7 | (-1.7: 0.2) | |  | | | Ref | | 0.7 | (-1.6: 0.3) | |

^1^ Adjusted for age group (<60, 60-69, 70-81 years), practice postcode quintile of index of multiple deprivation (IMD), and country (Scotland, Wales, Northern Ireland, England).

Ref = reference category; 95%CI = 95% Confidence interval.
